# The chromatin remodeling protein BRG1 regulates HSC-myofibroblast differentiation and liver fibrosis

**DOI:** 10.1038/s41419-023-06351-5

**Published:** 2023-12-14

**Authors:** Yuwen Zhu, Aoqi Kang, Yameng Kuai, Yan Guo, Xiulian Miao, Li Zhu, Ming Kong, Nan Li

**Affiliations:** 1 Key Laboratory of Targeted Intervention of Cardiovascular Disease and Collaborative Innovation Center for Cardiovascular Translational Medicine, Departments of Pathophysiology and Human Anatomy, Nanjing Medical University, Nanjing, China; 2 College of Life Sciences and Institute of Biomedical Research, Liaocheng University, Liaocheng, China; 3 Department of Hepatobiliary Surgery, The Third Affiliated Hospital of Soochow University, Changzhou, China; 4 State Key Laboratory of Natural Medicines, Department of Pharmacology, China Pharmaceutical University, Nanjing, China

**Keywords:** Liver fibrosis, Cell biology, Transcription

## Abstract

Excessive fibrogenic response in the liver disrupts normal hepatic anatomy and function heralding such end-stage liver diseases as hepatocellular carcinoma and cirrhosis. Myofibroblasts, derived primarily from hepatic stellate cells (HSCs), are the effector of liver fibrosis. In the present study we investigated the mechanism by which Brahma-related gene 1 (BRG1, encoded by *Smarca4*) regulates HSC-myofibroblast transition and the implication in intervention against liver fibrosis. We report that BRG1 expression was elevated during HSC maturation in cell culture, in animal models, and in human cirrhotic liver biopsy specimens. HSC-specific deletion of BRG1 attenuated liver fibrosis in several different animal models. In addition, BRG1 ablation in myofibroblasts ameliorated liver fibrosis. RNA-seq identified IGFBP5 as a novel target for BRG1. Over-expression of IGFBP5 partially rescued the deficiency in myofibroblast activation when BRG1 was depleted. On the contrary, IGFBP5 knockdown suppressed HSC-myofibroblast transition in vitro and mollified liver fibrosis in mice. Mechanistically, IGFBP5 interacted with Bat3 to stabilize the Bat3-TβR complex and sustain TGF-β signaling. In conclusion, our data provide compelling evidence that BRG1 is a pivotal regulator of liver fibrosis by programming HSC-myofibroblast transition.

## Introduction

Liver fibrosis, defined as the deposition of extracellular matrix (ECM) in the hepatic interstitia, is generally considered a host defense mechanism taking place during liver injuries. A process similar to wound healing, liver fibrosis prevents precipitous collapse of liver architecture and facilitates recovery of liver function [1]. Excessively strong scarring or failed resolution of fibrogenesis, however, often leads to irreversible remodeling of hepatic anatomy and irretrievable loss of liver function. Owing to the global pandemic of obesity and metabolic syndrome, patients with liver fibrosis, estimated to account for 5% of general population, have steadily increased in the past decade [2]. Without effective intervention, liver fibrosis precedes such devastating hepatic pathologies as cirrhosis and hepatcellular carcinoma and is inversely correlates with prognosis of end-stage liver diseases necessitating liver transplantation [3]. As such, liver fibrosis serves as a useful biomarker for diagnosis and a benchmark against which the efficacies of interventional regimens are evaluated. Despite extensive and rigorous research, there is currently no pharmacotherapy to treat liver fibrosis specifically and effectively.

Regardless of etiology, myofibroblasts are the major effector cell type producing large quantities of ECM proteins to mediate fibrogenic response in the liver [4]. The concept of myofibroblasts was first introduced by Gabbiani and Majno to describe a morphologically distinct cell population during dermal wound healing [5]. Myofibroblasts, possessing both fibroblast-like and muscle-like characteristics, are highly proliferative, migratory, and contractile. Myofibroblasts are absent from the quiescent liver but quickly emerge following liver injuries inflicted by hepatotoxins, cholestasis, pathogens, or excessive influx of nutrients. The origin from which myofibroblasts are derived during liver fibrosis has been a topic of constant debate and controversy. Recent breakthroughs in genetic lineage tracing techniques have been the driving force in the delineation of the hepatic myofibroblast pool. Mederacke et al. provide heretofore the most elegant and comprehensive fate-mapping data to show that hepatic stellate cells (HSCs), labeled by lecithin-retinol acyltransferase (*Lrat*) represent the predominant source of ECM-producing myofibroblasts in well-established models of liver fibrosis induced by injection with CCl_4_ or thioacetamide, bile duct ligation, or feeding with 3,5-diethoxycarbonyl-1,4-dihydro-collidin diet or methionine-and-choline deficient diet [6]. This conclusion has recently been challenged by Yang et al. in a study using single-cell RNA-seq technique, which reveals that portal fibroblasts (PFs), instead of HSCs, constitute the major source of myofibroblasts during early stages of cholestatic liver fibrosis [7]. A major caveat in the analyses by Mederacke et al. [6] and Yang et al. [7] is that neither study traced the dynamic evolution of myofibroblasts but instead focused on a single end-point to reach the conclusion. These discrepancies notwithstanding, HSCs likely contribute most significantly to the hepatic myofibroblast lineage in most, if not all, cases of liver fibrosis.

HSCs in quiescent state function as a reservoir of lipids and vitamin A. During trans-differentiation into myofibroblasts, HSCs undergo profound morphological and functional alterations accompanied by a concurrent shift in cellular transcriptome [8]. In mammalian cells, transcriptional events are intimately regulated by the epigenetic machinery divided into several branches including DNA and histone modifying enzymes, histone variants, non-coding regulatory RNAs, and chromatin remodeling proteins [9]. TGF-β is among the most potent stimuli that promote HSC-myofibroblast transition. In response to TGF-β stimulation, SMAD proteins become phosphorylated and migrate into the nucleus to program a pro-fibrogenic response, which coincidently is synonymous with remodeling of the chromatin structure [10]. Brahma related gene 1 (BRG1) is a conserved component of the SWI/SNF chromatin remodeling complex by providing the ATPase activity to drive nucleasome mobilization. In the present study we investigated the role of BRG1, a chromatin remodeling protein, in HSC-myofibroblast transition and liver fibrosis focusing on the underlying mechanism. Our data indicate that BRG1 is a pivotal regulator of liver fibrosis by programming HSC-myofibroblast transition.

## Methods

### Animals

All animal experiments were reviewed and approved by the Ethics Committee on Humane Treatment of Laboratory Animals of Nanjing Medical University and were performed in accordance with the ethical standards laid down in the 1964 Declaration of Helsinki and its later amendments. *Smarca4*^f/f^ mice [11], *Lrat*-Cre mice [6], and *Postn*-Cre^ERT2^ mice [12] have been described previously. Liver fibrosis was induced in mice by CCl_4_ injection (1.0 ml/kg body weight as 50% vol/vol, twice a week for 4 weeks), bile-duct ligation (BDL), thioacetamide (TAA) injection (100 mg/kg every other day for 14 days), or methionine-and-choline deficient (MCD) diet feeding (Research Diets, A02082002BR) for 6 weeks.

### Cell culture

Human immortalized hepatic stellate cells (LX-2, ATCC) were maintained in DMEM supplemented with 10% FBS. Primary hepatic stellate cells were isolated and maintained as previously described [13]. Primary human hepatic stellate cells were purchased from Lonza. Small interfering RNAs were purchased from Dharmacon. Adenovirus carrying FLAG-tagged IGFBP5 vector was purchased from GenePharma (Shanghai, China). Transient transfections were performed with Lipofectamine 2000. Luciferase activities were assayed 24–48 h after transfection using a luciferase reporter assay system (Promega) as previously described.

### Protein extraction, immunoprecipitation, and western blotting

Whole cell lysates were obtained by re-suspending cell pellets in RIPA buffer (50 mM Tris pH7.4, 150 mM NaCl, 1% Triton X-100) with freshly added protease inhibitor (Roche). Specific antibodies or pre-immune IgGs (P.I.I.) were added to and incubated with cell lysates overnight before being absorbed by Protein A/G-plus Agarose beads (Santa Cruz). Precipitated immune complex was released by boiling with 1X SDS electrophoresis sample buffer. Western blot analyses were performed with anti-α-SMA (Abcam, ab7817), anti-collagen type I (Proteintech, 14695-1), anti-BRG1 (Cell Signaling Techy, 49306), anti-IGFBP5 (Abcam, ab254324), anti-Bat3 (Proteintech, 26417-1), anti-TβRI (Abcam, ab235578), anti-TβRII (Abcam, ab184948), anti-SMAD1 (Proteintech, 10429-1), anti-phospho-SMAD1 (Cell Signaling Tech, 13820), anti-SMAD2 (Proteintech, 12570-1), anti-phospho-SMAD2 (Cell Signaling Tech, 18338), anti-SMAD3 (Proteintech, 661516-1), anti-phospho-SMAD3 (Cell Signaling Tech, 9520), and anti-β-actin (Sigma, A2228) antibodies.

### RNA isolation and real-time PCR

RNA was extracted with the RNeasy RNA isolation kit (Qiagen). Reverse transcriptase reactions were performed using a SuperScript First-strand Synthesis System (Invitrogen) as previously described [14–17]. Real-time PCR reactions were performed on an ABI Prism 7500 system with the following primers: for for mouse *Col1a1*, 5′-ATTTGAAGTCCCAGAAAG-3′ and 5′-AGAAACTCCCGTCTGCTC-3′; for mouse *Acta2*, 5′-CCTGTTTCGGGAGCAGAA-3′ and 5′-GGTTATATAGCCCCCTGG-3′; for mouse *Col3a1*, 5′-GACTCTGGCAAAACTCAAAGTATCA-3′ and 5′-TAGGAATGTGCTTTGTGATAGCCT-3′; for mouse *Lox*, 5′-ACGTTTCCAATCACATTACG-3′ and 5′-ACGGTCCTCCTCTCCCCTTT-3′; for mouse *Ctgf*, 5′-CTTCTGCGATTTCGGCTCC-3′ and 5′-TACACCGACCCACCGAAGA-3′; for mouse *Timp1*, 5′-CCAGAGCCGTCACTTTGCTT-3′ and 5′-AGGAAAAGTAGACAGTGTTCAGGCTT-3′. Ct values of target genes were normalized to the Ct values of housekeekping control gene (18 s, 5′-CGCGGTTCTATTTTGTTGGT-3′ and 5′-TCGTCTTCGAAACTCCGACT-3′ for both human and mouse genes) using the ΔΔCt method and expressed as relative mRNA expression levels compared to the control group which is arbitrarily set as 1.

### EdU incorporation assay

5-ethynyl-2′-deoxyuridine (EdU) incorporation assay was performed in triplicate wells with a commercially available kit (Thermo Fisher) as previously described [18]. Briefly, the EdU solution was diluted with the culture media and added to the cells for an incubation period of 2 h at 37 °C. After several washes with 1XPBS, the cells were then fixed with 4% formaldehyde and stained with Alexa Fluor™ 488. The nucleus was counter-stained with DAPI. The images were visualized by fluorescence microscopy and analyzed with Image-Pro Plus (Media Cybernetics). For each group, at least six different fields were randomly chosen and the positively stained cells were counted and divided by the number of total cells. The data are expressed as relative EdU staining compared to the control group arbitrarily set as 100%.

### RNA sequencing and data analysis

RNA-seq was performed and analyzed as previously described [19]. Total RNA was extracted using the TRIzol reagent according to the manufacturer’s protocol. RNA purity and quantification were evaluated using the NanoDrop 2000 spectrophotometer (Thermo Scientific, USA). RNA integrity was assessed using the Agilent 2100 Bioanalyzer (Agilent Technologies, Santa Clara, CA, USA). Then the libraries were constructed using TruSeq Stranded mRNA LT Sample Prep Kit (Illumina, San Diego, CA, USA) according to the manufacturer’s instructions and sequenced on an Illumina HiSeq X Ten platform and 150 bp paired-end reads were generated. Raw data (raw reads) of fastq format were firstly processed using Trimmomatic and the low quality reads were removed to obtain the clean reads. The clean reads were mapped to the mouse genome (Mus_musculus.GRCm38.99) using HISAT2. FPKM of each gene was calculated using Cufflinks, and the read counts of each gene were obtained by HTSeqcount. Differential expression analysis was performed using the DESeq (2012) R package. *P* value < 0.05 and fold change > 2 or fold change < 0.5 was set as the threshold for significantly differential expression. Hierarchical cluster analysis of differentially expressed genes (DEGs) was performed to demonstrate the expression pattern of genes in different groups and samples. GO enrichment and KEGG pathway enrichment analysis of DEGs were performed respectively using R based on the hypergeometric distribution.

### Human cirrhosis specimens

Liver biopsies were collected from patients with cirrhosis referring to Nanjing Drum Tower Hospital. Written informed consent was obtained from subjects or families of liver donors. All procedures that involved human samples were approved by the Ethics Committee of the Nanjing Drum Tower Hospital (approval reference #: 2020-155-01) and adhered to the principles outlined in the Declaration of Helsinki. Hepatic stellate cells and hepatocytes were isolated from the biopsy specimens as previously described [20].

### Statistical analysis

For comparison between two groups, two-tailed *t*-test was performed. For comparison among three or more groups, one-way ANOVA or two-way ANOVA with post-hoc Turkey analyses were performed using an SPSS package. The assumptions of normality were checked using Shapiro–Wilks test and equal variance was checked using Levene’s test; both were satisfied. *p* values smaller than 0.05 were considered statistically significant (*). All in vitro experiments were repeated at least three times and three replicates were estimated to provide 80% power.

## Results

### BRG1 expression correlates with hepatic stellate cell activation

**Fig. 1:**
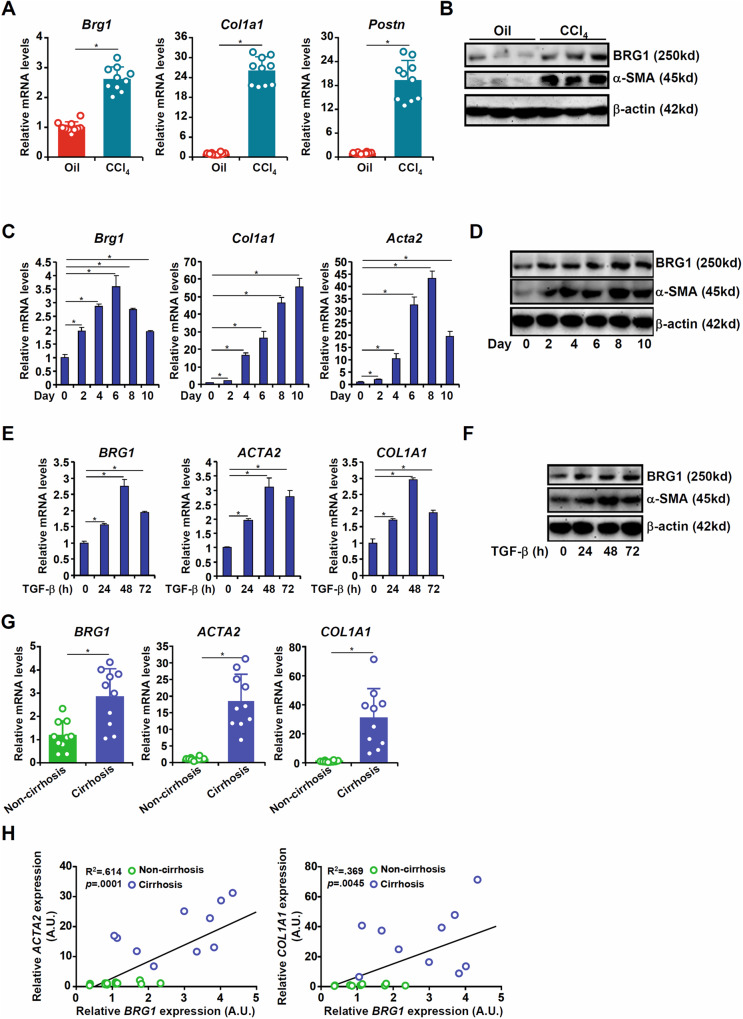
BRG1 expression correlates with hepatic stellate cell activation. **A, B** C57/B6 mice were injected with CCl_4_ to induce liver fibrosis as described in Methods. Primary HSCs were isolated and gene expression was examined by qPCR and Western blotting. *N* = 10 mice fore each group. **C, D** Primary HSCs were isolated from C57/B6 mice and underwent spontaneous activation in vitro. Gene expression was examined by qPCR and Western blotting. **E, F** LX-2 cells were treated with or without TGF-β (5 ng/ml). Cells were harvested at indicated time points and gene expression was examined by qPCR and Western blotting. **G, H** HSCs were isolated from human liver biopsy specimens and gene expression was examined by qPCR. *N* = 10 cases for each group. Linear regression was performed using Graphpad. Data are expressed as the means ± SD. *p < 0.05.

In order to determine whether BRG1 expression levels might be correlated with HSC-myofibroblast transition, C57/B6 mice were injected with CCl_4_ to induce liver fibrosis. Primary HSCs isolated from CCl_4_-injected mice displayed elevated levels of myofibroblast marker genes compared to those isolated from vehicle-injected mice; coincidently, BRG1 expression was higher in the activated HSCs (myofibroblasts) than the quiescent HSCs (Fig. 1A, B). Similar observations were made in two alternative models of liver fibrosis: in both the BDL model (Fig. S1) and the MCD model (Fig. S2), BRG1 expression was up-regulated during HSC-myofibroblast transition in vivo. Next, quiescent HSCs isolated from C57/B6 mice underwent spontaneous activation in vitro: levels of myofibroblast marker genes were progressively up-regulated along with that of BRG1 as HSCs transitioned to myofibroblasts (Fig. 1C, D). When the human immortalized HSCs (LX-2) were exposed to TGF-β, BRG1 expression again was augmented along with myofibroblast marker genes (Fig. 1E, F). Finally, primary HSCs isolated from patients with cirrhosis expressed higher levels of BRG1 than those from healthy individuals (Fig. 1G). Importantly, a significant positive correlation between BRG1 and myofibroblast marker genes was identified in humans (Fig. 1H).

### Hepatic stellate cell specific manipulation of BRG1 alters fibrogenic response in mice

**Fig. 2:**
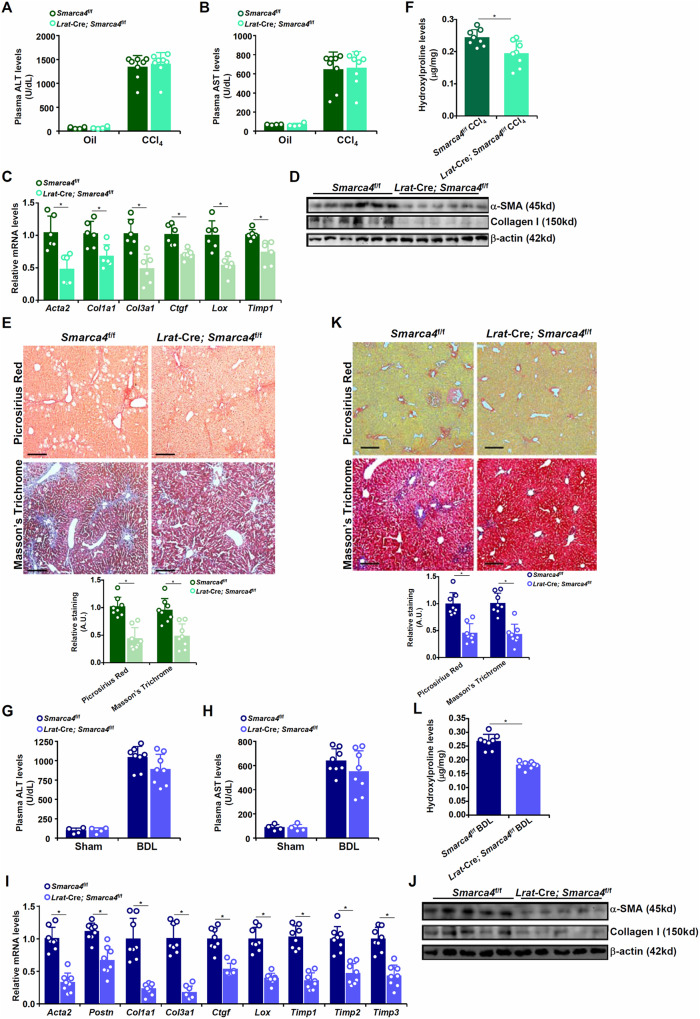
Hepatic stellate cell specific deletion of BRG1 attenuates liver fibrosis in mice. **A–F** 8-week male *Smarca4*^*f*/*f*^; *Lrat*-Cre mice and *Smarca4*^*f*/*f*^ mice were subjected to CCl_4_ injection for 4 wk. Plasma ALT (**A**) and AST (**B**) levels. Expression levels of pro-fibrogenic genes were examined by qPCR (**C**) and Western blotting (**D**). Picrosirius red and Masson’s trichrome staining (**E**). Hydroxylproline quantification (**F**). **G–L** 8-week male *Smarca4*^*f*/*f*^; *Lrat*-Cre and *Smarca4*^*f*/*f*^ mice were subjected to BDL for 2 wk. Plasma ALT (**G**) and AST (**H**) levels. Expression levels of pro-fibrogenic genes were examined by qPCR (**I**) and Western blotting (**J**). Picrosirius red and Masson’s trichrome staining (**K**). Hydroxylproline quantification (**L**). *N* = 4–8 mice for each group. Data are expressed as the means ± SD. *p < 0.05.

Next, the *Lrat*-Cre strain [6] was used to drive HSC-specific gene manipulation of BRG1. When crossed with the *Smarca4*^f/f^ mice, BRG1 was deleted in HSCs but not in hepatocytes as verified by Western blotting (Fig. S3). HSC-specific BRG1 deletion did not influence liver injury when the mice were subjected to CCl_4_ injection as evidenced by comparable plasma ALT (Fig. 2A) and AST (Fig. 2B) levels. QPCR (Fig. 2C) and Western blotting (Fig. 2D) revealed that BRG1 deletion down-regulated the expression of myofibroblast marker genes in the liver. Histological staining (Fig. 2E) and hydroxylproline quantification (Fig. 2F) confirmed that BRG1 deletion resulted in a reduction of collagenous tissue deposition in the liver following CCl_4_ injection. Similar observations were made in the BDL model (Fig. 2G–L), the TAA model (Fig. S4), and the MCD model (Fig. S5) that HSC-specific BRG1 deletion dampened liver fibrosis.

### Myofibroblast-specific BRG1 deletion attenuates liver fibrosis in mice

**Fig. 3:**
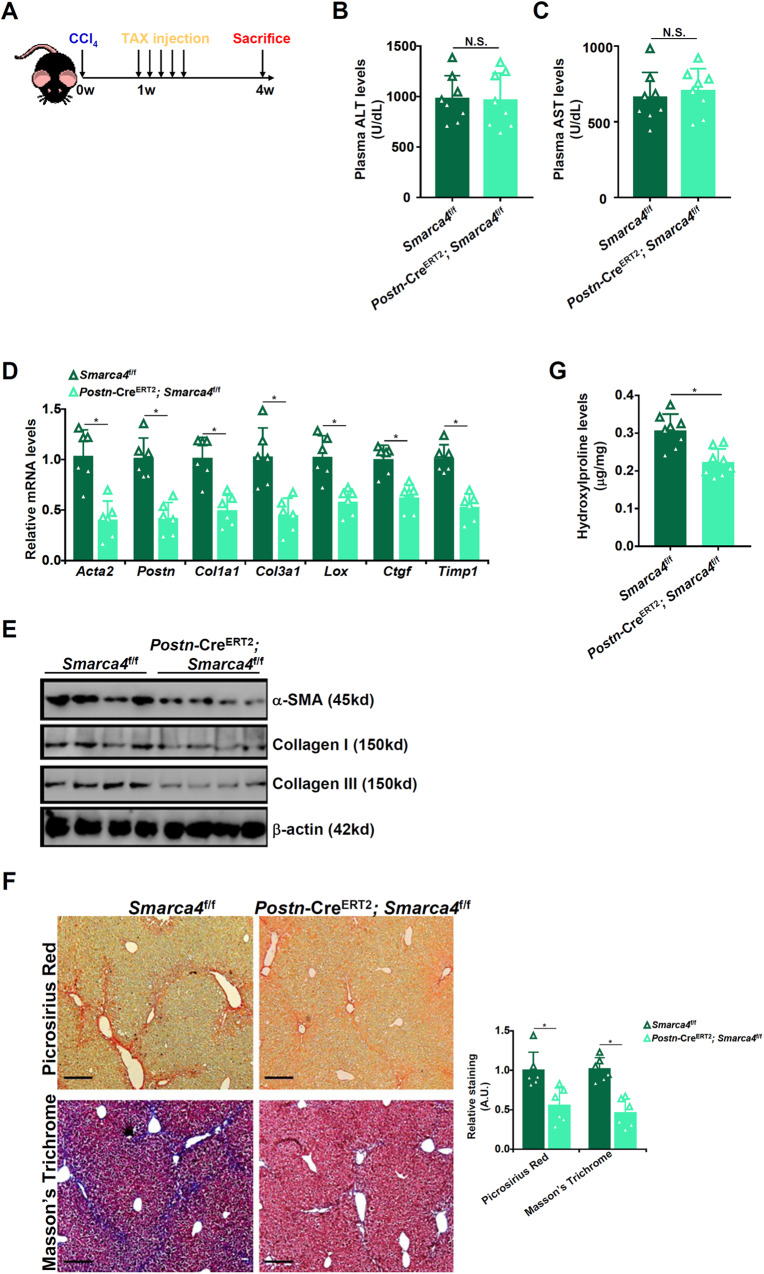
Myofibroblast-specific BRG1 deletion attenuates liver fibrosis in mice. The *Postn*-Cre^ERT2^; *Smarca4*^f/f^ mice and the *Smarca4*^f/f^ mice were subjected to CCl_4_ injection followed by tamoxifen injection. **A** Scheme of protocol. **B** Plasma ALT levels. **C** Plasma AST levels. **D**, **E** Expression levels of pro-fibrogenic genes were examined by qPCR and Western. **F** Picrosirius red and Masson’s trichrome staining. **G** Hydroxylproline quantification. *N* = 8 mice for each group. Data are expressed as the means ± SD. *p < 0.05.

Regardless of origin, myofibroblasts are the ultimate cell type that mediates liver fibrosis. We asked whether targeting BRG1 in Postn^+^ myofibroblasts would influence liver fibrosis. To this end, the *Postn*-Cre^ERT2^ mice were crossed to the *Smarca4*^f/f^ mice. Both the *Smarca4*^f/f^ mice and the *Postn*-Cre^ERT2^; *Smarca4*^f/f^ mice were subjected to CCl_4_ injection; one week later, the mice received five consecutive injections of tamoxifen to allow myofibroblast-specific BRG1 deletion (Fig. 3A). Indistinguishable levels of liver injury were observed in the *Postn*-Cre^ERT2^; *Smarca4*^f/f^ mice and the *Smarca4*^f/f^ mice (Fig. 3B, C). Of interest, the *Postn*-Cre^ERT2^; *Smarca4*^f/f^ mice displayed significant reduction of myofibroblast marker gene expression compared to the control mice (Fig. 3D, E). Concordantly, there were fewer collagenous tissues in the *Postn*-Cre^ERT2^; *Smarca4*^f/f^ livers than in the control livers (Fig. 3F, G). In the BDL model, post-injury deletion of BRG1 in myofibroblasts similarly attenuated liver fibrosis (Fig. S6). Combined, these data suggest that BRG1 might be a key regulator of HSC-myofibroblast transition in vivo.

### RNA-seq identifies IGFBP5 as a novel target for BRG1

**Fig. 4:**
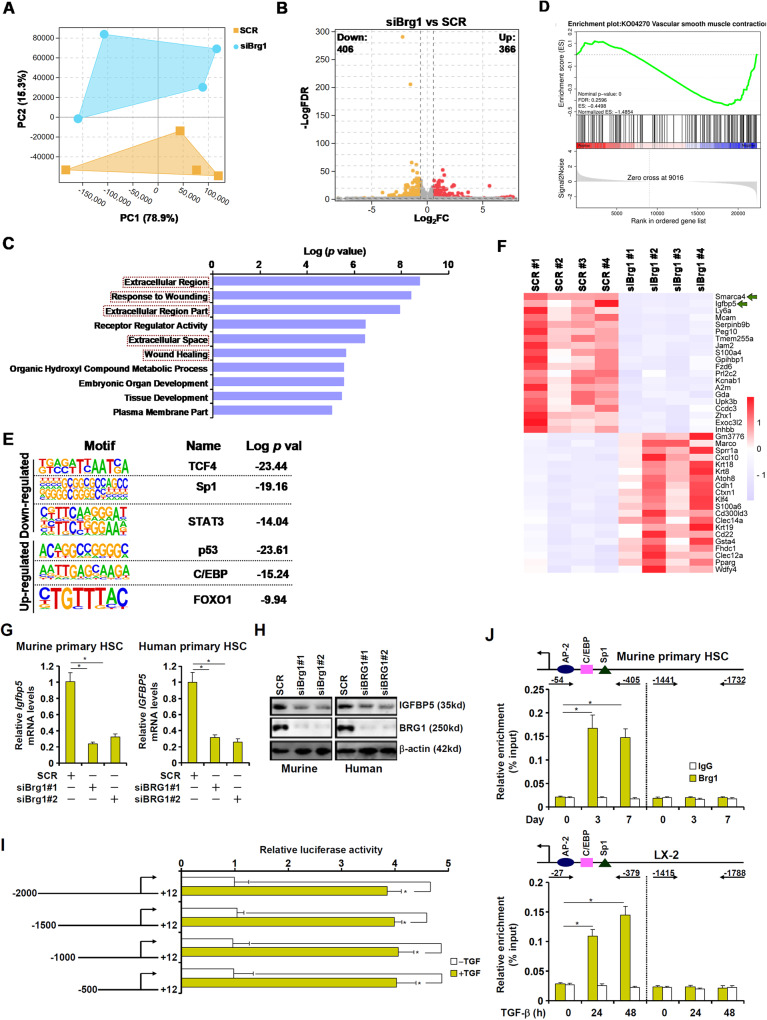
RNA-seq identifies IGFBP5 as a novel target for BRG1. **A–E** Primary murine HSCs were transfected with siRNA targeting BRG1 or scrambled siRNA (SCR). RNA-seq was performed as described in Methods. (**A**) Principal component (PC) plot. (**B**) Volcano plot. (**C**) GO analysis. (**D**) Geneset enrichment analysis (GSEA) analysis. (**E**) Heatmap. **F, G** Primary human/murine HSCs were transfected with siRNA targeting BRG1 or scrambled siRNA (SCR). IGFBP5 expression levels were examined by qPCR and Western blotting. **H** IGFBP5 promoter constructs were transfected into LX-2 cells followed by treatment with TGF-β (2 ng/ml) for 24 h. Luciferase activities were normalized by GFP fluorescence and protein concentration. **I** ChIP assays were performed in primary murine HSCs or LX-2 cells treated with TGF-β. **J** Murine or human primary HSCs were transfected with indicated siRNAs followed by transduction with lentivirus carrying an IGFBP5 expression vector. Myofibroblast marker gene expression levels were examined by qPCR. Data are expressed as the means ± SD. *p < 0.05.

To identify the downstream target of BRG1 during HSC-myofibroblast transition, experiments were performed to compare the transcriptome of primary murine HSCs in which BRG1 was depleted by siRNAs. BRG1 depletion down-regulated myofibroblast marker gene expression and attenuated cell proliferation (Fig. S7). RNA-seq showed that BRG1 depletion markedly altered cellular transcriptome (Fig. 4A). Using 1.5xfold change and FDR < 0.05 as a cut-off, we detected 772 differentially expressed genes (DEGs) with slightly more genes being down-regulated (406) than up-regulated (366) as a result of BRG1 depletion (Fig. 4B). GO analysis (Fig. 4C) and geneset enrichment analysis (Fig. 4D) both indicated that pathways related to myofibroblast maturation, including extracellular matrix remodeling, cell proliferation, and acquisition of muscle-like contraction, were acutely affected by BRG1 depletion. HOMER analysis revealed that BRG1 deficiency impacted the activities of several well-documented transcription factors involved in tissue fibrosis (Fig. 4E).

Insulin-like growth factor binding protein 5 (IGFBP5) was the top ranked (by FDR) gene affected by BRG1 depletion (Fig. 4F). IGFBP5 expression was up-regulated in spontaneously activated murine HSCs (Fig. S8) and in TGF-β treated LX-2 cells (Fig. S9). In addition, in vivo activated HSCs displayed higher IGFBP5 expression than quiescent HSCs (Fig. S10). BRG1 knockdown decreased IGFBP5 expression in both murine and human primary HSCs in vitro (Fig. 4G, H). BRG1 deletion in HSCs down-regulated whereas BRG1 over-expression in HSCs up–regulated IGFBP5 expression in vivo in different models of liver fibrosis (Fig. S11). Moreover, a positive correlation between BRG1 expression and IGFBP5 expression was identified in human cirrhosis specimens (Fig. S12). Importantly, immunofluorescence staining showed a co-expression of BRG1 and IGFBP5 in Col1^+^ myofibroblasts in the cirrhotic liver specimens (Fig. S13).

Reporter assay indicated that a TGF response element might be located between −500 and +12 of the IGFBP5 promoter (Fig. 4I). ChIP assay confirmed that BRG1 occupancy on the proximal IGFBP5 promoter, but not the distal IGFBP5 promoter, was enhanced during HSC-myofibroblast transition (Fig. 4J). Over-expression of IGFBP5 in HSCs depleted of BRG1 partially rescued the deficiency of HSC-myofibroblast transition (Fig. S14). Together, these data suggest that BRG1 might program HSC-myofibroblast transition and liver fibrosis by activating IGFBP5 transcription.

### IGFBP5 is essential for HSC-myofibroblast transition in vitro and liver fibrosis in vivo

**Fig. 5:**
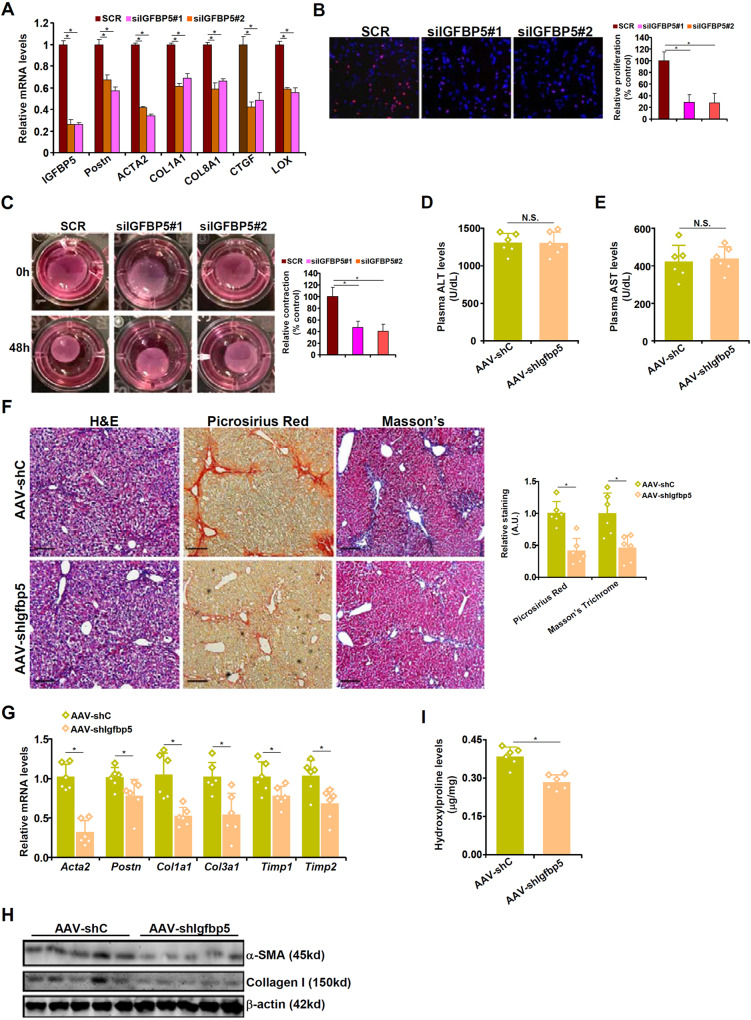
IGFBP5 is essential for HSC-myofibroblast transition in vitro and liver fibrosis in vivo. **A–D** Primary human HSCs were transfected with siRNAs targeting IGFBP5 or scrambled siRNA (SCR). Myofibroblast marker genes were examined by qPCR (**A**). Cell proliferation was evaluated by EdU incorporation (**B**). Collagen contraction assay (**C**). **D–I** C57/B6 mice were injected with AAV6 carrying shRNA under the control of the *Postn* promoter followed by injection with CCl_4_ for 4 wk. Plasma ALT (**D**) and AST (**E**) levels. H&E staining, picrosirius red staining, and Masson’s trichrome staining (**F**). Myofibroblast marker gene expression levels were examined by qPCR (**G**) and Western blotting (**H**). Hydroxylproline quantification (**I**). *N* = 6 mice for each group. Data are expressed as the means ± SD. *p < 0.05.

The next series of experiments were performed to determine the role of IGFBP5 in HSC-myofibroblast transition and liver fibrosis. IGFBP5 knockdown weakened the acquisition of myofibroblast phenotype in human (Fig. 5A–C) and murine (Fig. S15) HSCs as assayed by qPCR, EdU incorporation, and collagen contraction. Next, shRNA targeting IGFBP5 was placed downstream of the *Postn* promoter and packaged into an AAV6 viral vector [21]. C57/B6 mice were injected via tail vein the shIgfbp5 virus or the control virus followed by CCl_4_ injection to induce liver fibrosis. QPCR revealed that IGFBP5 levels were down-regulated in myofibroblasts but not in hepatocytes (Fig. S16). Immunofluorescence staining further verified that IGFBP5 was knocked down in Col1^+^ myofibroblasts in the liver (Fig. S17). Myofibroblast-specific IGFBP5 deletion did not alter liver injury as evidenced by comparable plasma ALT (Fig. 5D) and AST (Fig. 5E) levels. However, liver fibrosis was significantly dampened in the mice injected with the shIgfbp5 virus compared to the mice injected with the control virus (Fig. 5F–I). The efficacy of targeting IGFBP5 in the intervention of liver fibrosis was further verified in the BDL model (Fig. S18). In addition, a positive correlation between IGFBP5 and myofibroblast marker gene levels was identified in human cirrhosis specimens (Fig. S19).

### IGFBP5 promotes TGF-β signaling by stabilizing the TβR-Bat3 interaction

**Fig. 6:**
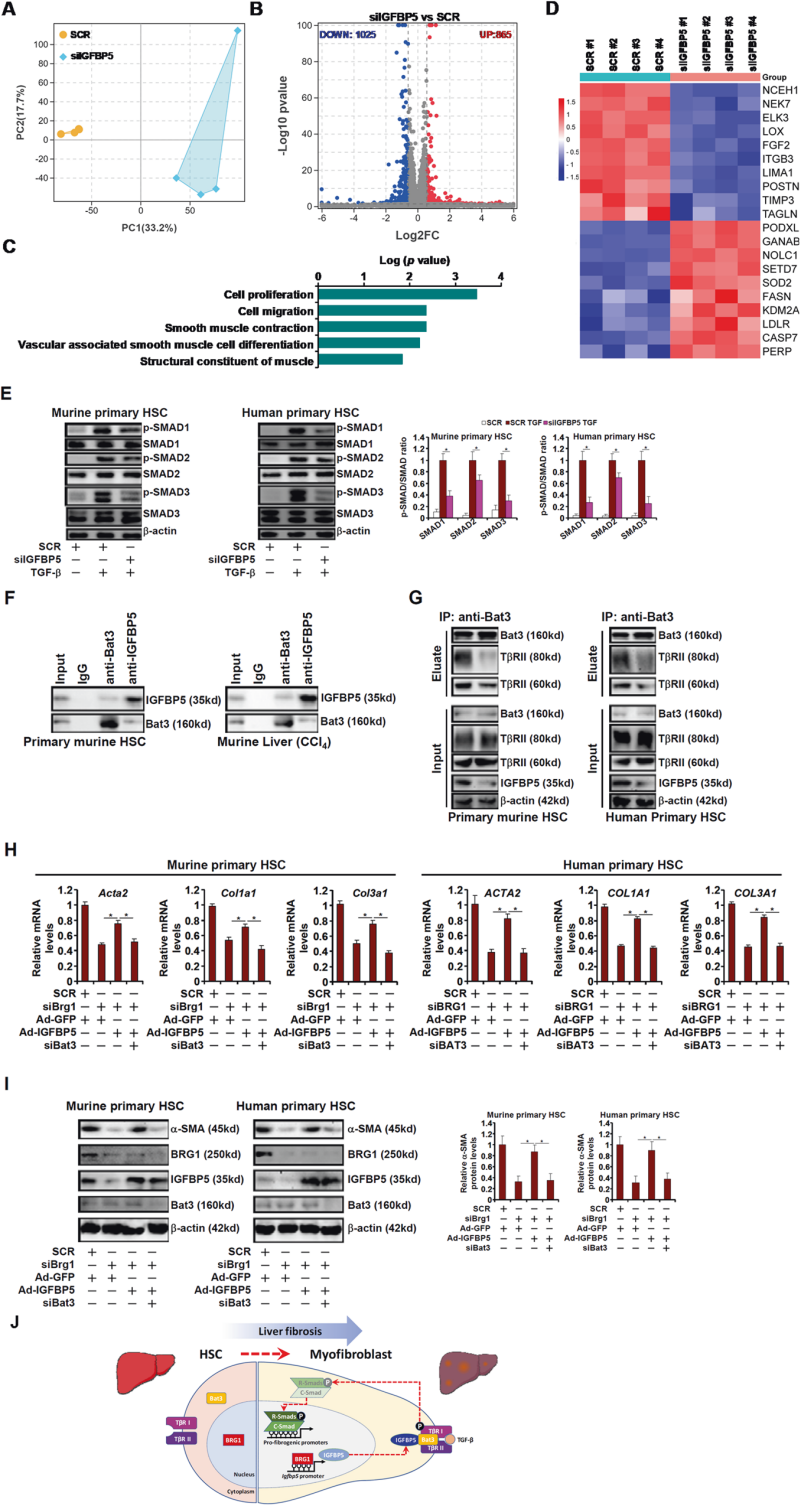
IGFBP5 promotes TGF-β signaling by stabilizing the TβR-Bat3 interaction. **A–D** Primary human HSCs were transfected with siRNA targeting IGFBP5 or scrambled siRNA (SCR). RNA-seq was performed as described in Methods. PCA plot (**A**). Volcano plot (**B**). GESA analysis (**C**). Heatmap (**D**). **E** Primary murine and human HSCs were transfected with indicated siRNAs followed by TGF-β stimulation for 15 min. SMAD phosphorylation was examined by Western blotting. **F** Whole lysates from primary murine HSCs or murine livers following CCl_4_ injection were used for immunoprecipitation with indicated antibodies. **G** Primary HSCs were transfected with indicated siRNAs followed by TGF-β stimulation for 15 min. Immunoprecipitation was performed with anti-Bat3. **H, I** Primary murine and human HSCs were transfected with siRNAs targeting BRG1, BAT3, or scrambled siRNA (SCR) and transduced with IGFBP5 adenovirus. Myofibroblast marker gene expression was examined by qPCR and Western blotting. Data are expressed as the means ± SD. *p < 0.05. **J** A schematic model.

RNA-seq assay indicated that IGFBP5 deficiency in HSCs led to significant overhaul of the cellular transcriptome (Fig. 6A, B). GO analysis showed that IGFBP5 deficiency preferentially affected genes involved in cell proliferation, migration, and muscle-like contraction, all of which contribute to HSC-myofibroblast transition (Fig. 6C). Among the top differentially expressed genes were well-documented myofibroblast markers such as Periostin (encoded by *POSTN*), Transgelin (encoded by *TAGLN*), and Lysyl oxidase (encoded by *LOX*), all of which were down-regulated by IGFBP5 knockdown (Fig. 6D).

It was noted that IGFBP5 knockdown markedly inhibited TGF-β signaling, the master regulator of myofibroblast maturation, as evidenced by the dampening of SMAD1/2/3 phosphorylation in both murine and human primary HSCs (Fig. 6E). BioGRID is an online curator of comprehensive protein-protein interaction data (https://thebiogrid.org/). 112 unique IGFBP5-interactors in humans and 5 unique IGFBP5-interactors in mice have been identified; Bat3 (also known as Bag6) was the only overlapping IGFBP5-interactor in both species. The interaction between Bat3 and IGFBP5 was confirmed in cultured HSCs and in liver tissue lysates (Fig. 6F). Choi et al. have previously reported that Bat3 contributes to collagen type I transcription in mesangial cells by interacting with TGF-β receptors [22]. We hypothesized that IGFBP5 might interact with Bat3 to stabilize the TβR-Bat3 complex thus sustaining TGF-β signaling. Indeed, co-immunoprecipitation assay showed that when IGFBP5 was knocked down the interaction between Bat3 and the TGF-β receptors became significantly weaker (Fig. 6G). More importantly, Bat3 depletion negated the ability of IGFBP5 over-expression, achieved through adenoviral transduction (see Fig. S20 for validation of over-experssion), to restore deficiency of the myofibroblast phenotype in the absence of BRG1 (Fig. 6H, I).

## Discussion

Aberrant fibrogenic response in the liver leads to the loss of hepatic anatomy and function contributing to the mortality of patients with end-stage liver diseases. Herein we describe a novel transcription mechanism whereby the chromatin remodeling protein BRG1 plays a pivotal role in HSC-myofibroblast transition and liver fibrosis. Consistent with a previously study by Li et al. showing that BRG1 drives HSC activation by interacting with SMAD3 [23], we show here that HSC-specific BRG1 deletion attenuates liver fibrosis in multiple different animal models. More importantly, mice with deletion of BRG1 from the *Postn*^+^ mature myofibroblast lineage phenocopy the HSC-conditional BRG1 deletion mice in models of liver fibrosis. These observations not only point to an unequivocal role for BRG1 in regulating the myofibroblast phenotype but allude to the previously established doctrine that HSCs represent a major, if not predominant, source for the pool of myofibroblasts during liver fibrosis. However, these data do not foreclose the possibility that BRG1 in other intrahepatic and extrahepatic cell lineages may similarly contribute to liver fibrosis. Of interest, Bu and colleagues have recently presented evidence to show that selective deletion of BRG1 in the CK19^+^ progenitor cell compartment ameliorates liver fibrosis and cholangiocarcinoma in mice [24]. Because both genetic lineage tracing [6] and single-cell RNA-seq [7] have excluded CK19^+^ cells as a meaningful origin of mature myofibroblasts, it was proposed that BRG1 might contribute to liver fibrosis by promoting expansion of these progenitor cells. Alternatively, it has been reported that BRG1 deletion in sinusoidal endothelial cells alleviates liver fibrosis by stimulating eNOS activity to increase NO bioavailability [25]. In addition, BRG1 in different hepatic cell compartments have been shown to cultivating a pro-pathogenic milieu by promoting ROS production and immune cell trafficking [26, 27]. Whereas these pitfalls do not at all dampen the essentiality of BRG1 in liver fibrosis, it is clear that more studies are needed to define cell-specific mechanisms for BRG1-dependent liver fibrosis.

Through transcriptomic analysis, it is discovered that BRG1 programs HSC-myofibroblast transition by affecting distinct sequence-specific transcription factors. On the one hand, BRG1 appears to able to augment the activities of well-established pro-fibrogenic transcription factors including TCF4 [28], Sp1 [29], and STAT3 [30]. On the other hand, BRG1 may suppress transcription factors involved in cell senescence/apoptosis (e.g., p53 and FOXO1) and de-activation of HSCs (e.g, C/EBP). Intriguingly, IGFBP5 is found to be most significantly altered by BRG1 deficiency, able to rescue myofibroblast phenotypes from BRG1 deficiency, and correlative/causal to liver fibrosis. Curiously, IGFBP5 has a relatively well-documented role in pulmonary fibrosis but not in fibrogenesis of other major organs (e.g., the liver). Consistent with our finding, Huang et al. have previously reported that IGFBP5, along with the myofibroblast marker periostin, is among a panel of molecules whose levels are sensitive to antifibrotic regiments in a precision-cut rat liver slice model [31]. Various studies have suggested that IGFBP5 likely contributes to fibrosis by promoting migroproliferative behaviors [32], stimulating ROS production [33], and prolonging survival [34] of ECM-producing myofibroblasts. We show here that IGFBP5 interacts with Bat3 to regulate TGF-β signaling, the most prominent pathway involved in tissue fibrosis. Alternatively, it has been observed that IGFBP5 can regulate cytoskeletal reorganization by inducing filamin a (FLNa) dephosphorylation and subsequent cleavage [35]. Cleaved FLNa then binds to SMADs to facilitate their nuclear translocation thus activating the TGF-β pathway. Moreover, IGFBP5 can translocate into the nucleus and function as a de novo transcription factor with a trans-activation domain being mapped to its N-terminus [36]. Of note, IGFBP5 has been detected to co-localize in the nuclei with vitamin D receptor (VDR) and repress VDR activity presumably by interfering with VDR-RXR heterodimerization [37]. Because VDR possesses potent antifibrotic activities in the liver [10], it is tempting to speculate that IGFBP5 might regulate liver fibrosis by functioning as an inhibitor of VDR. Contradictorily, there is evidence to show that IGFBP5 without the nuclear localization signal (NLS) acts as a stronger promoter of cellular migroproliferative behaviors than the wild type counterpart [38]. Additional investigation is warranted to clearly delineate the mechanism whereby IGFBP5 regulates HSC-myofibroblast transition.

In summary, our data provide compelling evidence that BRG1 is a pivotal regulator of liver fibrosis by programming HSC-myofibroblast transition. Despite the advances made by this report, outstanding questions remain. First, how does BRG1 influence epigenetic landscape and chromatin accessibility to coordinate the binding of transcription factors and basal transcription machinery during HSC-myofibroblast transition? Second, what are the sub-cellular compartment-specific roles of IGFBP5 in regulating HSC-myofibroblast transition? Third, can IGFBP5 be targeted by small-molecule compounds? Future studies should focus on the solving these lingering issues to generate safe and effective therapeutic strategies for the treatment of aberrant liver fibrosis.

### Supplementary information

Reporting Summar

Original Data File

online data

## Data Availability

The data that support the findings of this study are available upon reasonable request.
