# Supplementary material for: The chromatin remodeling protein BRG1 regulates HSC-myofibroblast differentiation and liver fibrosis
Source: Cell Death Dis. 2023 Dec 14;14(12):826. doi: 10.1038/s41419-023-06351-5 (PMC10719330; doi:10.1038/s41419-023-06351-5)
Supplement: Supplementary file 2 — Original Data File [file 41419_2023_6351_MOESM2_ESM.pdf]

Figure 1B

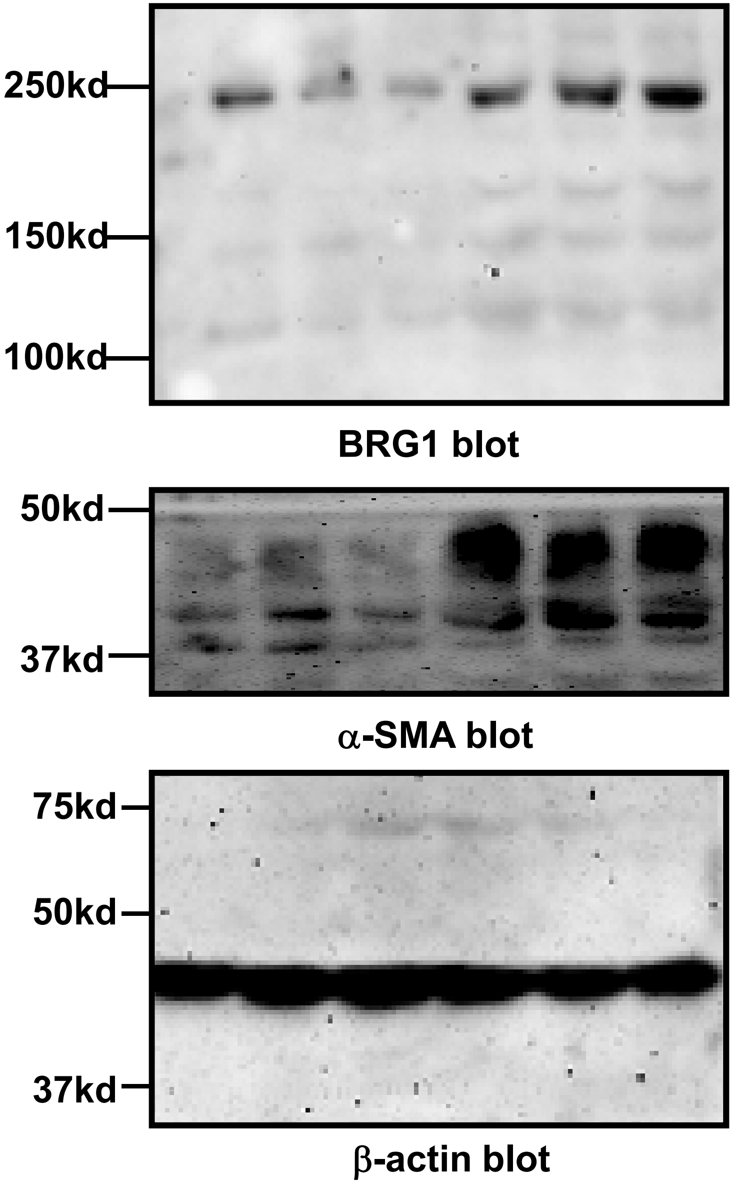

Figure 1D

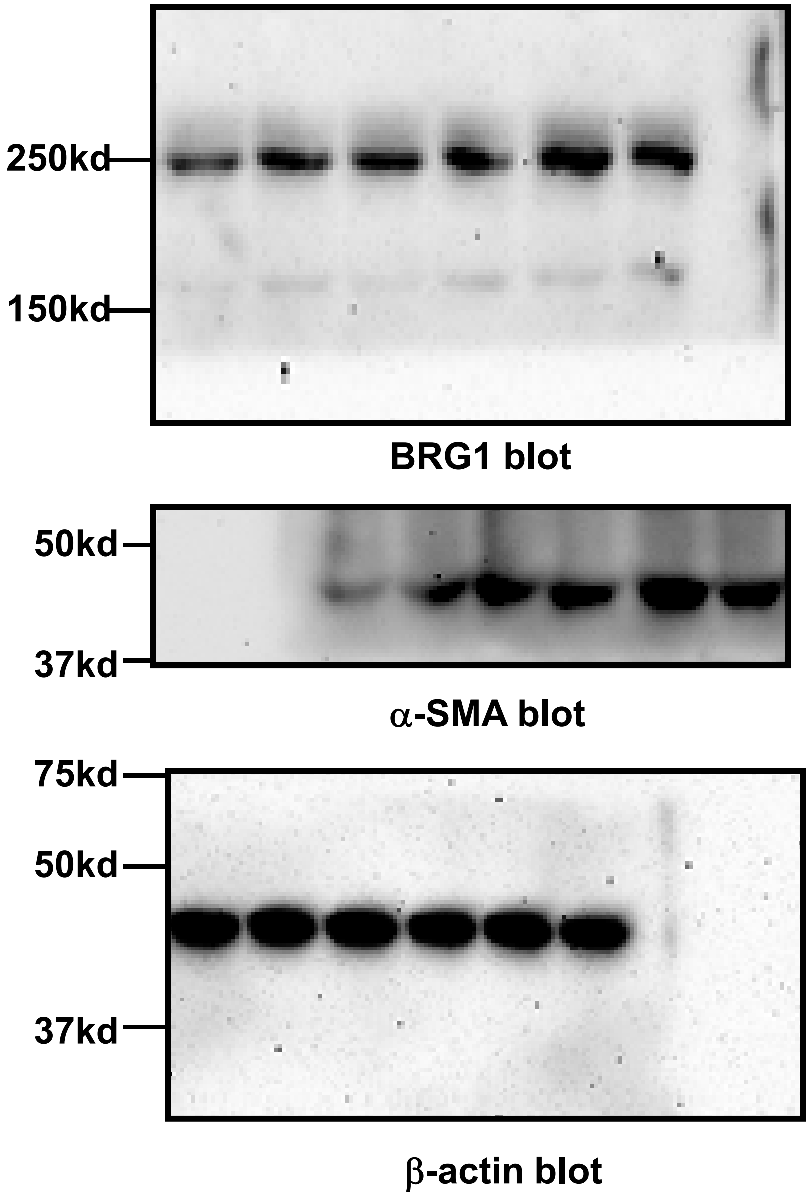

Figure 1F

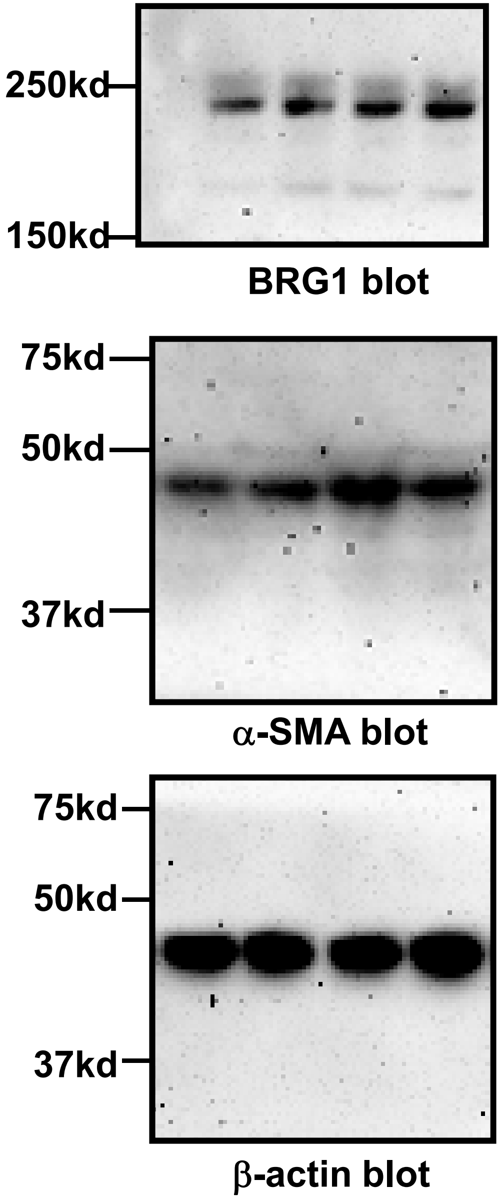

Figure 2D

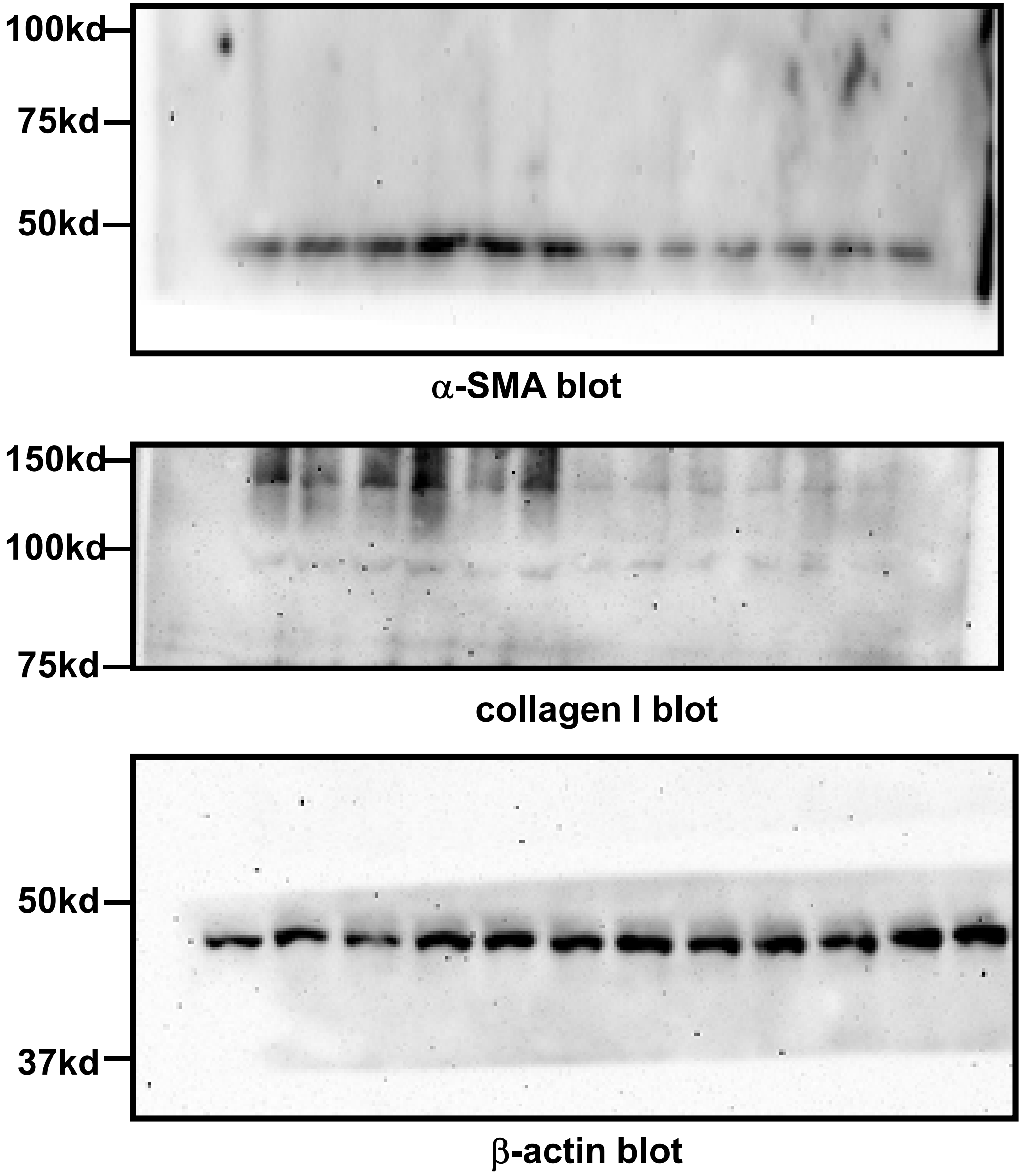

Figure 2J

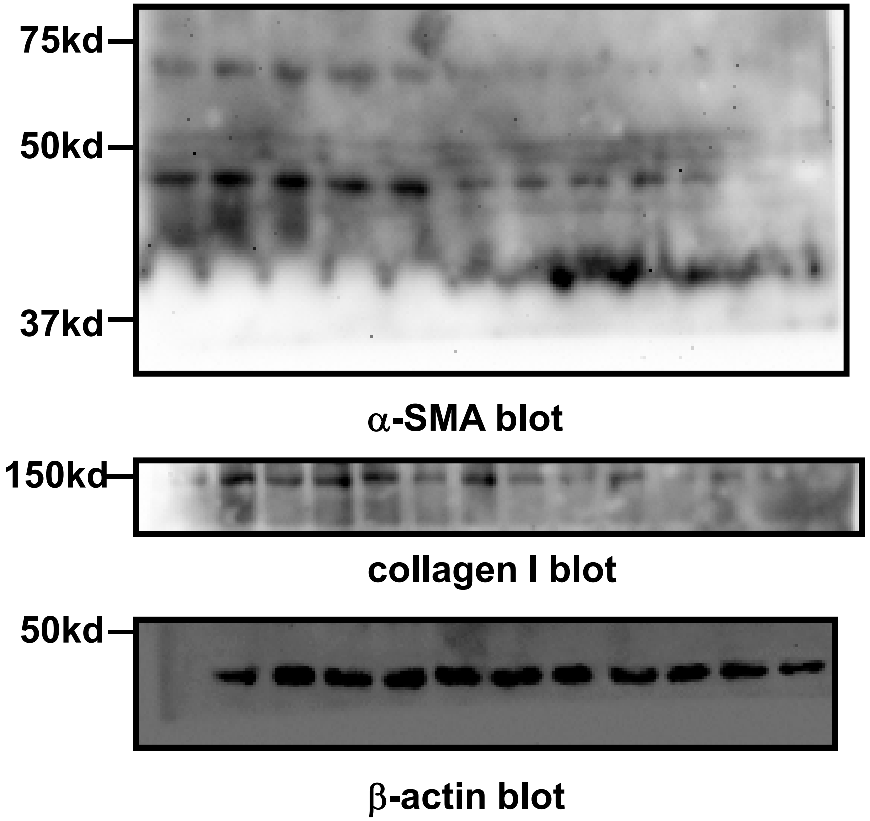
75kd
50kd
37kd
α-SMA blot
150kd
collagen I blot
50kd
β-actin blot

Figure 3E

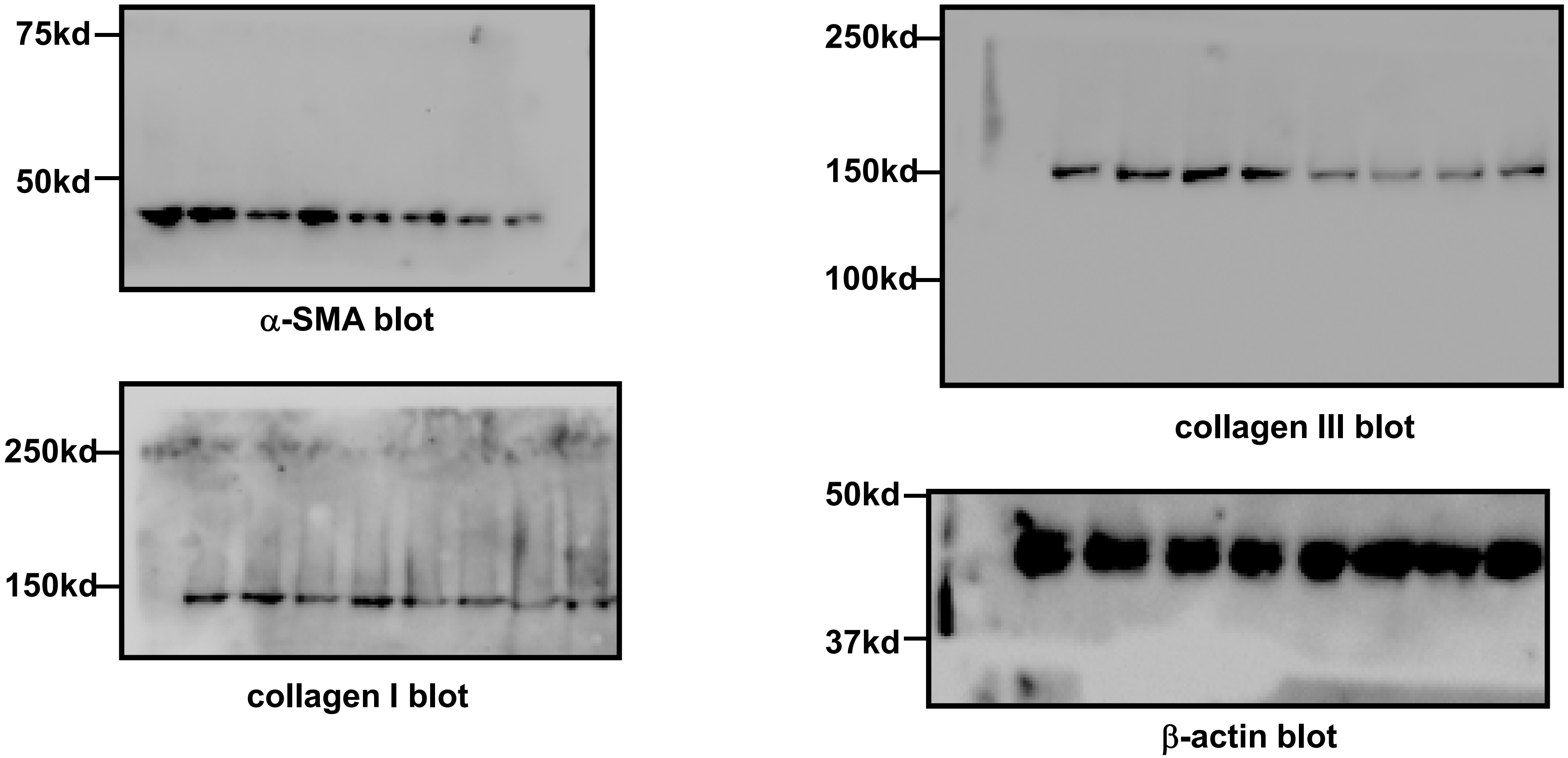
75kd
50kd
α-SMA blot
250kd
150kd
collagen I blot
250kd
150kd
100kd
collagen III blot
50kd
37kd
β-actin blot

Figure 4H

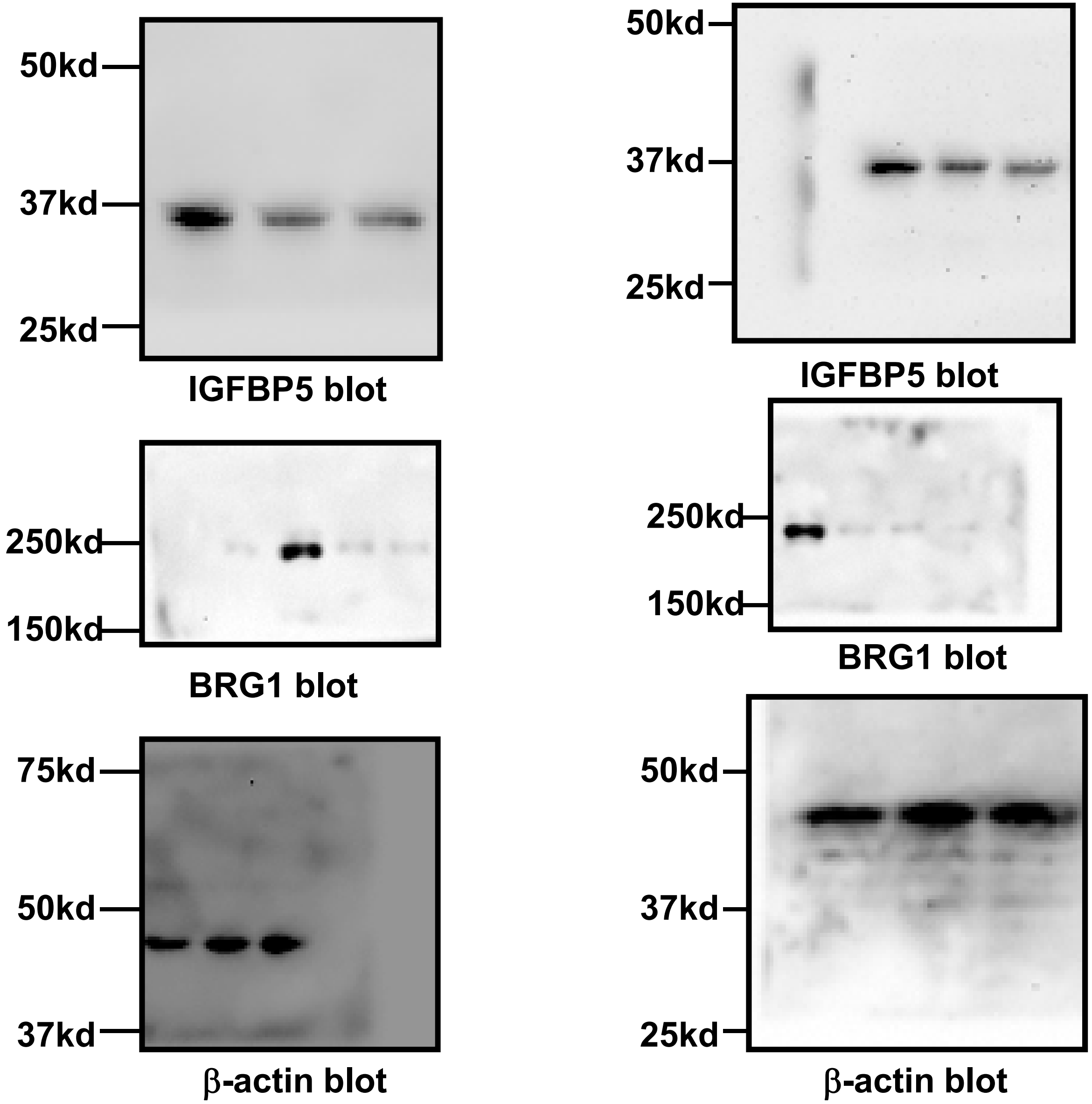
50kd
37kd
25kd
IGFBP5 blot
250kd
150kd
BRG1 blot
75kd
50kd
37kd
β-actin blot
50kd
37kd
25kd
IGFBP5 blot
250kd
150kd
BRG1 blot
50kd
37kd
25kd
β-actin blot

Figure 5H

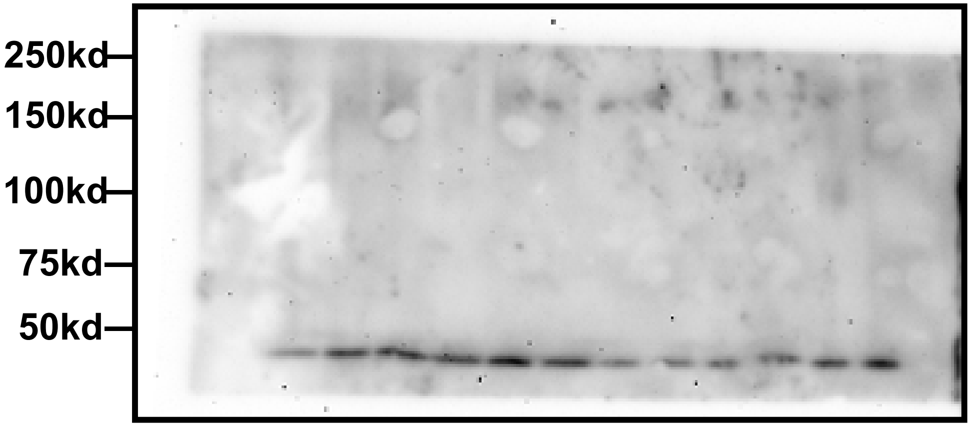

α-SMA blot

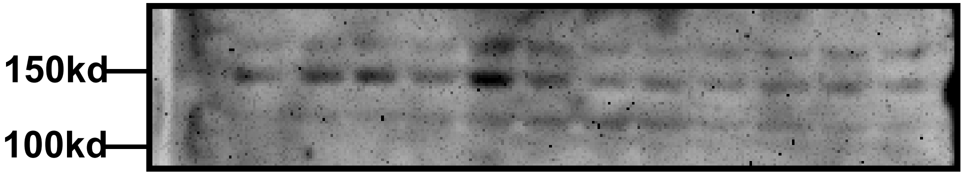

collagen I blot

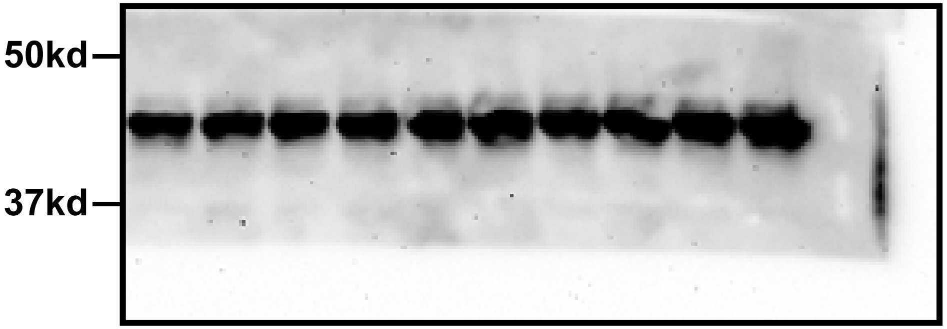

β-actin blot

Figure 6E

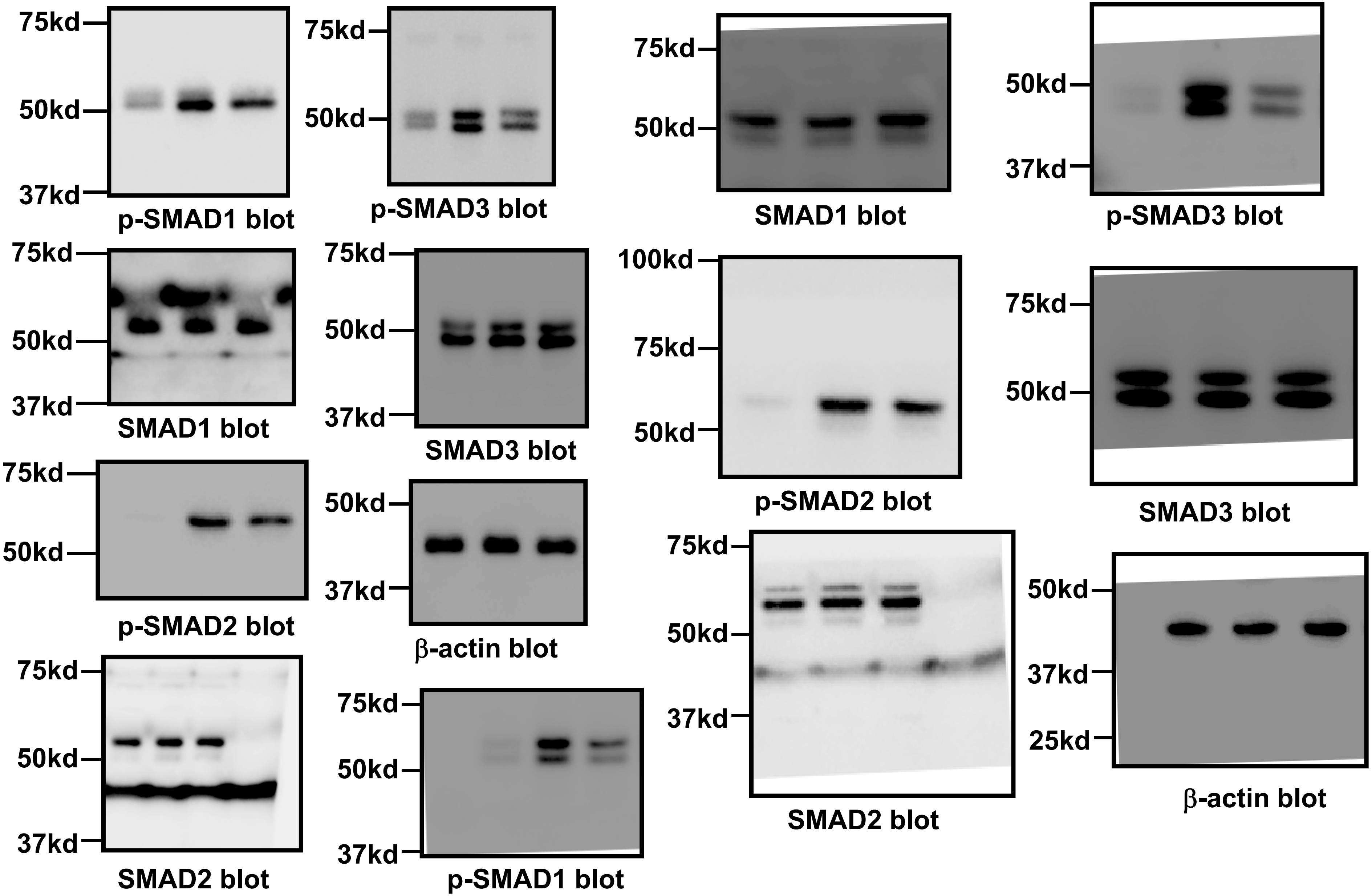
75kd
50kd
37kd
p-SMAD1 blot
75kd
50kd
p-SMAD3 blot
75kd
50kd
SMAD1 blot
50kd
37kd
p-SMAD3 blot
75kd
50kd
37kd
SMAD1 blot
75kd
50kd
37kd
SMAD3 blot
100kd
75kd
50kd
p-SMAD2 blot
75kd
50kd
SMAD3 blot
75kd
50kd
p-SMAD2 blot
50kd
37kd
β-actin blot
75kd
50kd
37kd
SMAD2 blot
50kd
37kd
25kd
β-actin blot
75kd
50kd
37kd
SMAD2 blot
75kd
50kd
37kd
p-SMAD1 blot

Figure 6F

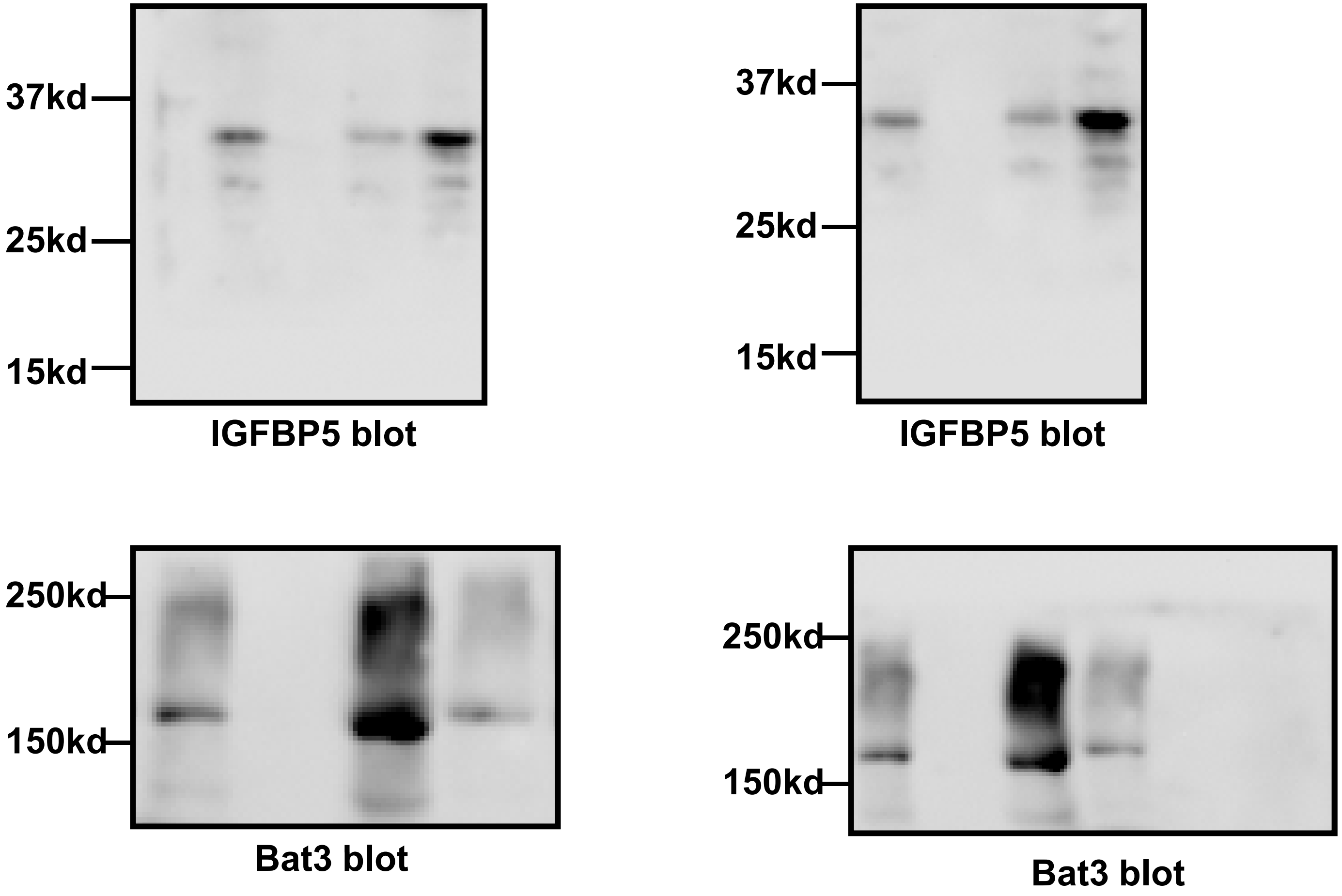
37kd
25kd
15kd
IGFBP5 blot
37kd
25kd
15kd
IGFBP5 blot
250kd
150kd
Bat3 blot
250kd
150kd
Bat3 blot

Figure 6G

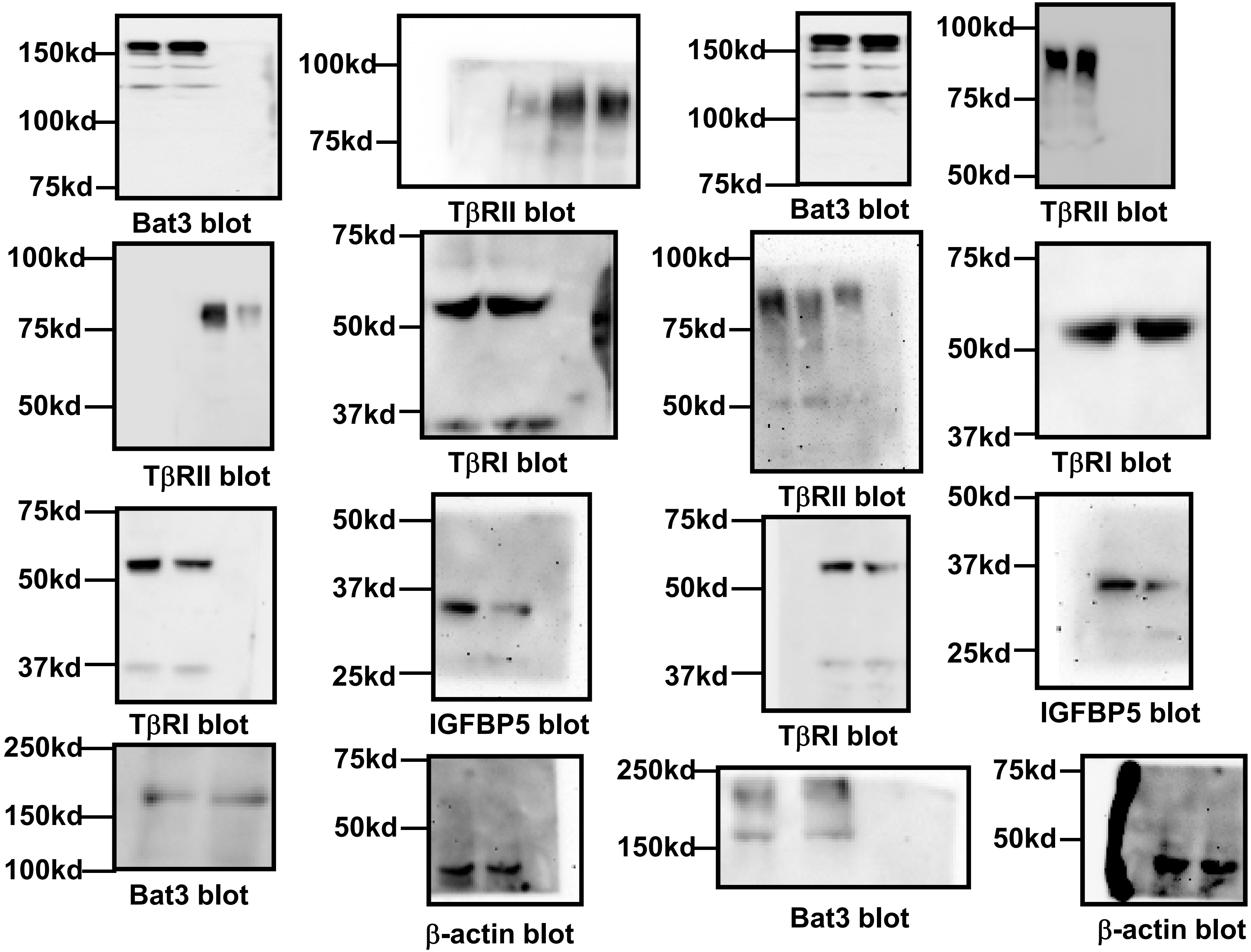
150kd
100kd
75kd
Bat3 blot
100kd
75kd
TβRII blot
150kd
100kd
75kd
Bat3 blot
100kd
75kd
50kd
TβRII blot
100kd
75kd
50kd
TβRII blot
75kd
50kd
37kd
TβRI blot
100kd
75kd
50kd
TβRII blot
75kd
50kd
37kd
TβRI blot
75kd
50kd
37kd
TβRI blot
50kd
37kd
25kd
IGFBP5 blot
75kd
50kd
37kd
TβRI blot
50kd
37kd
25kd
IGFBP5 blot
250kd
150kd
100kd
Bat3 blot
75kd
50kd
β-actin blot
250kd
150kd
Bat3 blot
75kd
50kd
β-actin blot

Figure 6I

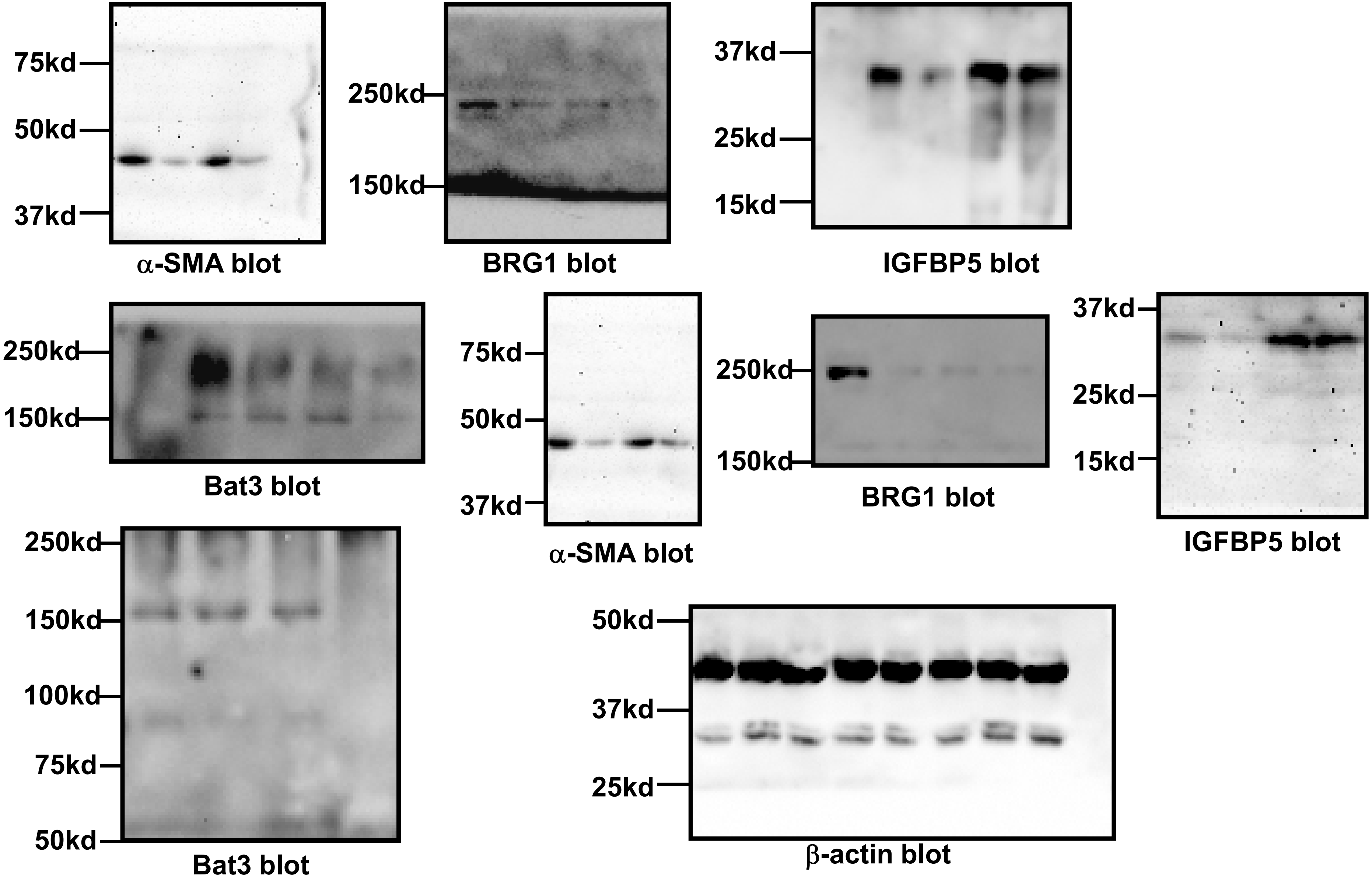

75kd
50kd
37kd
α-SMA blot
250kd
150kd
BRG1 blot
37kd
25kd
15kd
IGFBP5 blot
250kd
150kd
Bat3 blot
75kd
50kd
37kd
α-SMA blot
250kd
150kd
BRG1 blot
37kd
25kd
15kd
IGFBP5 blot
250kd
150kd
100kd
75kd
50kd
Bat3 blot
50kd
37kd
25kd
β-actin blot
