# Supplementary material for: The chromatin remodeling protein BRG1 regulates HSC-myofibroblast differentiation and liver fibrosis
Source: Cell Death Dis. 2023 Dec 14;14(12):826. doi: 10.1038/s41419-023-06351-5 (PMC10719330; doi:10.1038/s41419-023-06351-5)
Supplement: Supplementary file 3 — online data [file 41419_2023_6351_MOESM3_ESM.pdf]

Zhu YW et al: The chromatin remodeling protein BRG1 regulates HSC-myofibroblast differentiation and liver fibrosis
Online supplementary material
Supplementary figures: 20

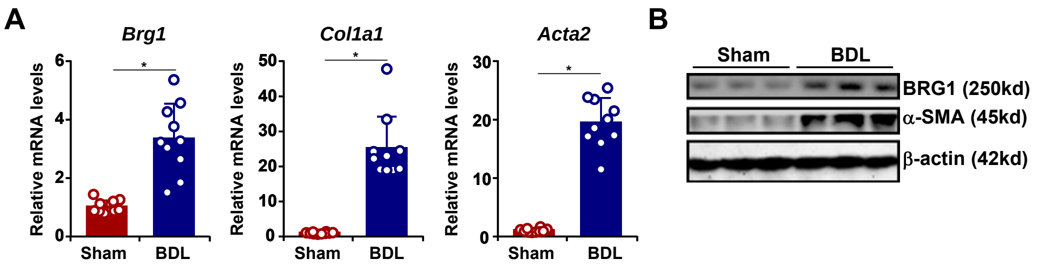

Fig.S1: (**A, B**) C57/B6 mice were subjected to the BDL procedure to induce liver fibrosis as described in Methods. Primary HSCs were isolated and gene expression was examined by qPCR and Western blotting.

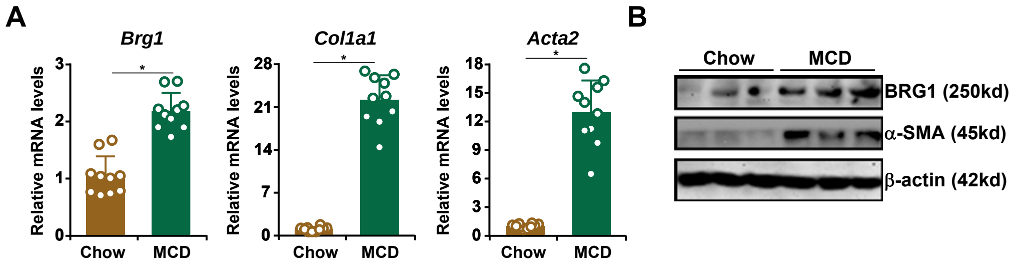

Fig.S2: (**A, B**) C57/B6 mice were fed a MCD diet to induce liver fibrosis as described in Methods. Primary HSCs were isolated and gene expression was examined by qPCR and Western blotting.

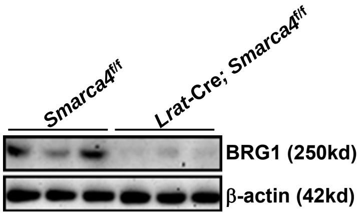

Fig.S3: Primary HSCs were isolated from Sm*arca4*$^{f/f}$; *Lrat*-Cre mice and *Smarca4*$^{f/f}$ mice and BRG1 expression was examined by Western blotting.

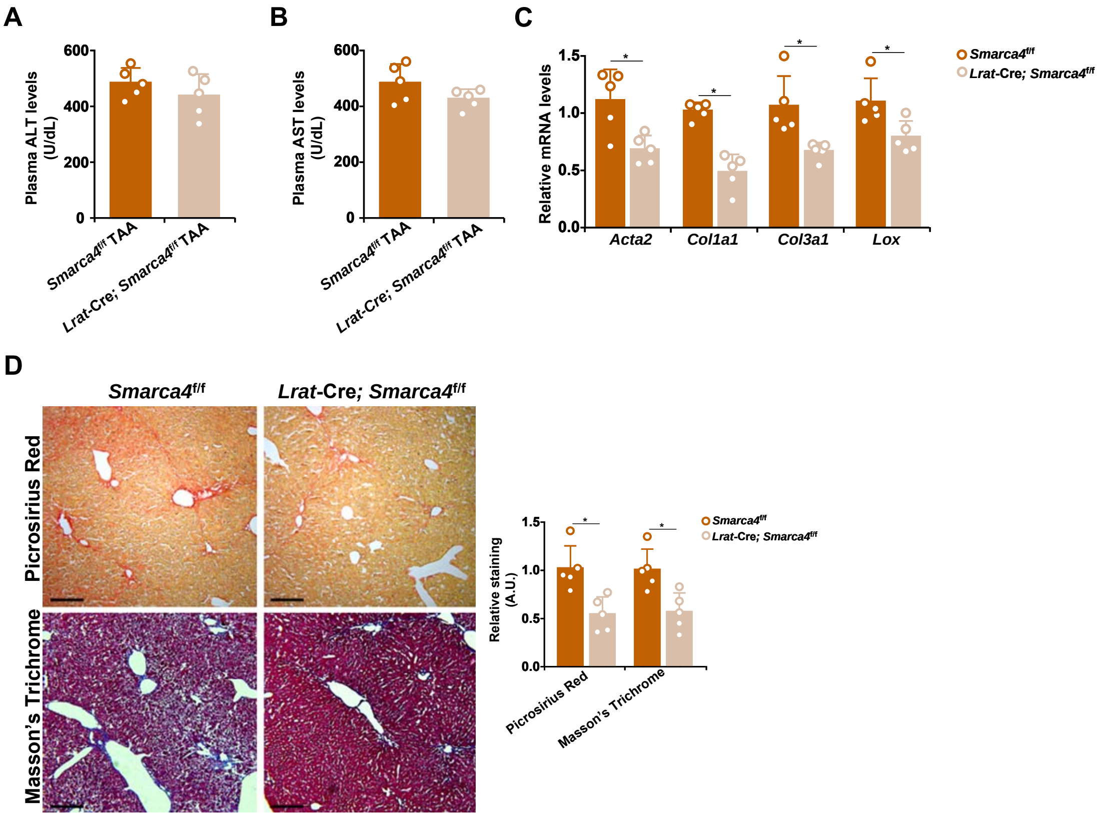

Fig.S4: (**A-D**) 8-week male *Smarca4*$^{f/f}$; *Lrat*-Cre and *Smarca4*$^{f/f}$ mice were subjected to TAA injection for 2 wk. Plasma ALT (A) and AST (B) levels. Expression levels of pro-fibrogenic genes were examined by qPCR (C). Picrosirius red and Masson's trichrome staining (D).

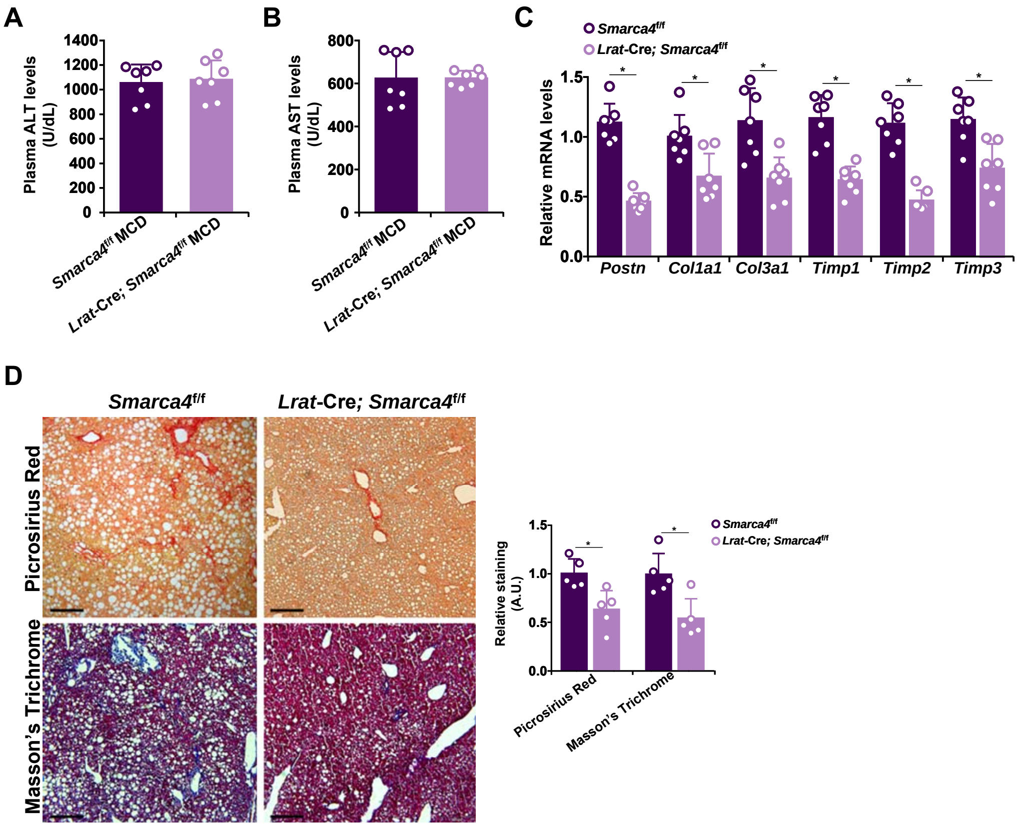

Fig.S5: (**A-D**) 8-week male *Smarca4*$^{f/f}$; *Lrat*-Cre and *Smarca4*$^{f/f}$ mice were fed the MCD diet for 6 wk. Plasma ALT (A) and AST (B) levels. Expression levels of pro-fibrogenic genes were examined by qPCR (C). Picrosirius red and Masson's trichrome staining (D).

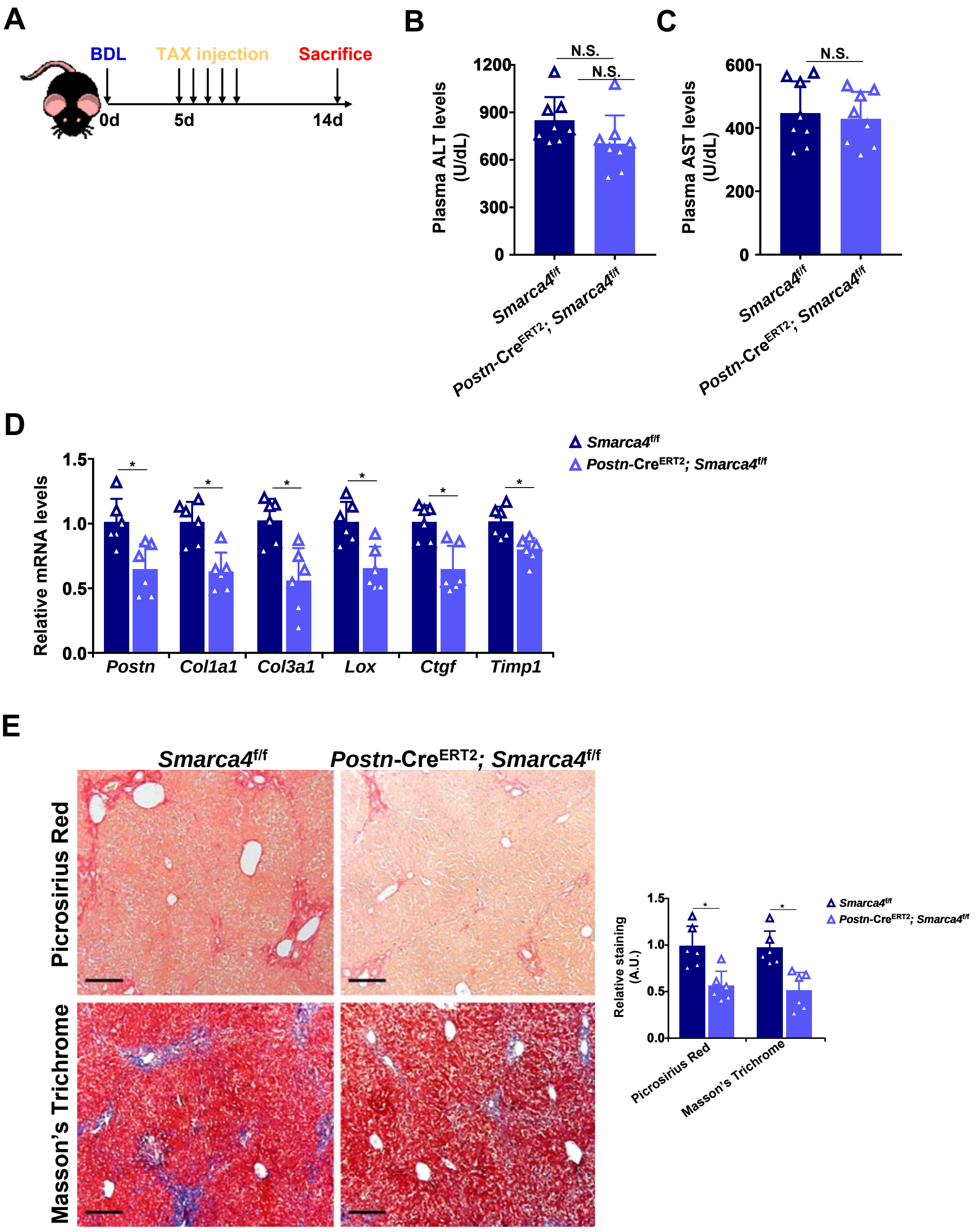

Fig.S6: The *Postn*-Cre^{ERT2}; *Smarca4*$^{f/f}$ mice and the *Smarca4*$^{f/f}$ mice were subjected to the BDL procedure followed by tamoxifen injection. (**A**) Scheme of protocol. (**B**) Plasma ALT levels. (**C**) Plasma AST levels. (**D**) Expression levels of pro-fibrogenic genes were examined by qPCR. (**E**) Picrosirius red and Masson's trichrome staining.

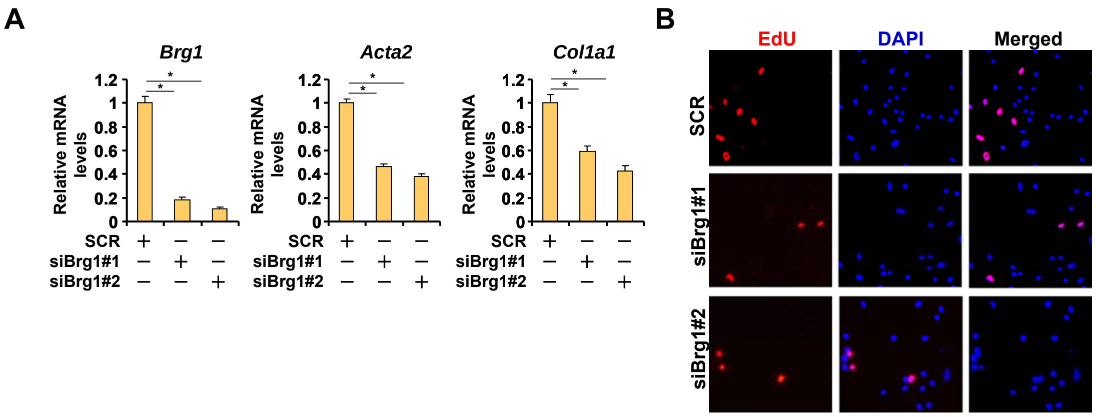

Fig.S7: (**A**) Primary murine HSCs were transfected with indicated siRNAs. Myofibroblast marker expression was examined by qPCR. (**B**) Primary murine HSCs were transfected with indicated siRNAs. Cell proliferation was measured by EdU incorporation.

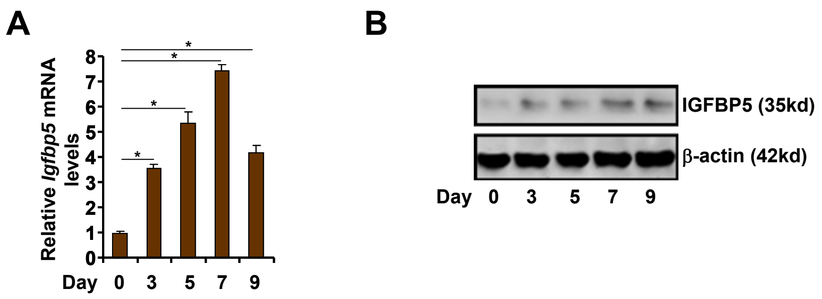

Fig.S8: (**A, B**) Primary murine HSCs were isolated and allowed to undergo spontaneous activation. IGFBP5 levels were examined by qPCR and Western blotting.

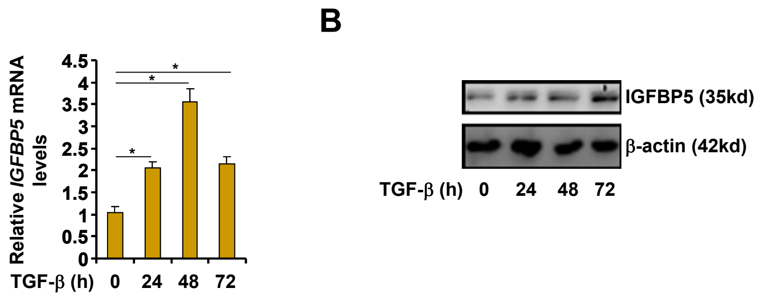

Fig.S9: (**A, B**) LX-2 cells treated with TGF-β (2ng/ml) and harvested at indicated time points. IGFBP5 levels were examined by qPCR and Western blotting.

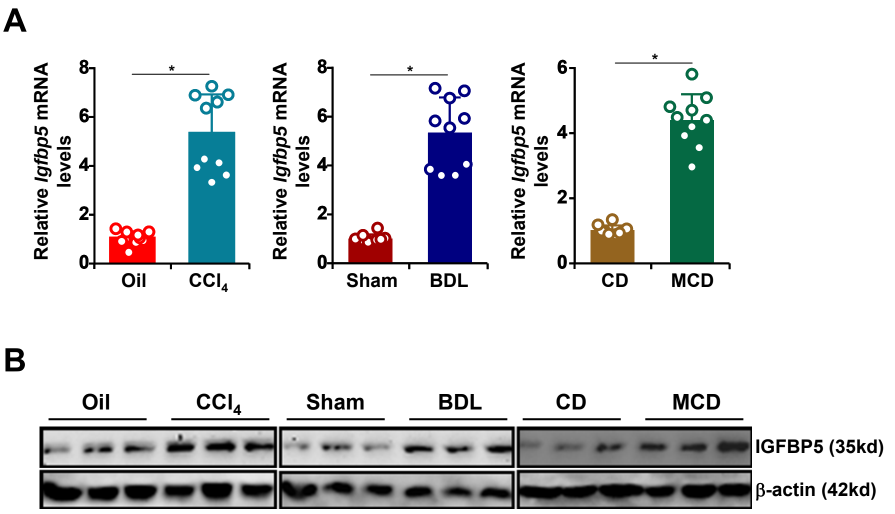

Fig.S10: (**A, B**) C57/B6 mice were injected with CCl_4, or subjected to the BDL procedure, or fed the MCD diet to induce liver fibrosis as described in Methods. Primary HSCs were isolated and IGFBP5 expression was examined by qPCR and Western blotting.

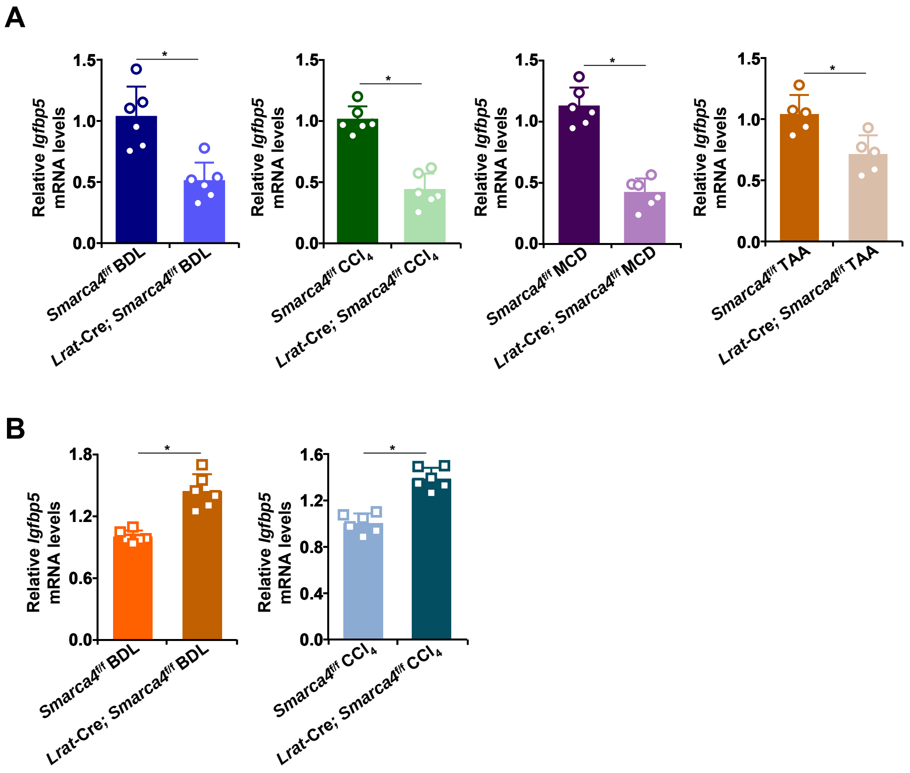

Fig.S11: (**A**) The *Smarca4*$^{f/f}$; *Lrat*-Cre and *Smarca4*$^{f/f}$ mice were subjected to CCl_4 injection, or the BDL procedure, or TAA injection, or MCD feeding. IGFBP5 expression was examined by qPCR. (**B**) The Rosa$^{Smarca4/+}$; *Lrat*-Cre and Rosa$^{Smarca4/+}$ mice were subjected to CCl_4 injection, or the BDL procedure. IGFBP5 expression was examined by qPCR.

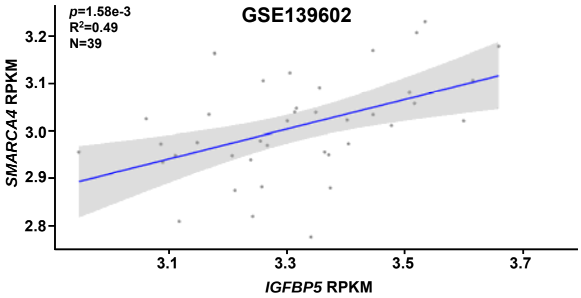

Fig.S12: Expression data for BRG1 and IGFBP5 in human cirrhosis specimens were extracted from publicly deposited dataset (GSE139602). Linear correlation was performed with Graphpad.

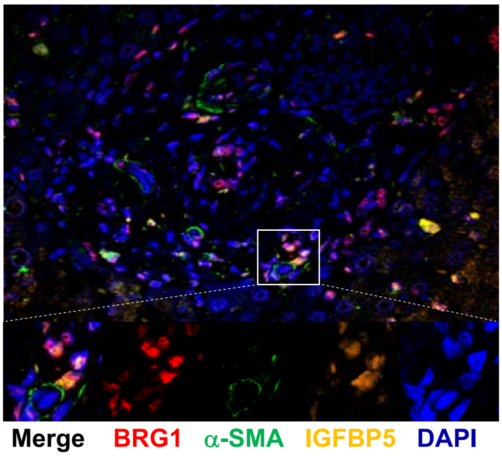

Fig.S13: Detection of BRG1 and IGFBP5 in human cirrhotic liver specimen by immunofluorescence staining using indicated antibodies.

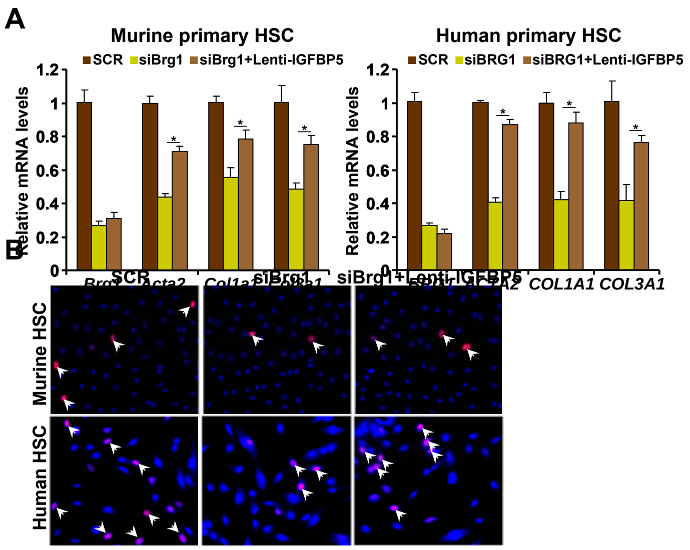

Fig.S14: (**A, B**) Primary murine and human HSCs were transfected with indicated siRNAs followed by transduction with lentivirus carrying an IGFBP5 over-expression vector. Myofibroblast marker expression was examined by qPCR. Cell proliferation was measured by EdU incorporation.

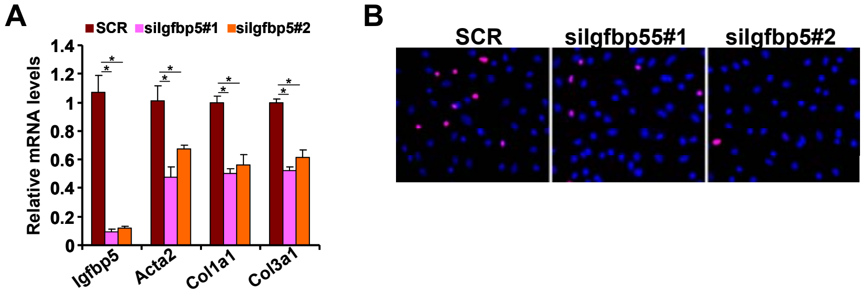

Fig.S15: (**A, B**) Primary murine HSCs were transfected with indicated siRNAs. Myofibroblast marker expression was examined by qPCR. Cell proliferation was measured by EdU incorporation.

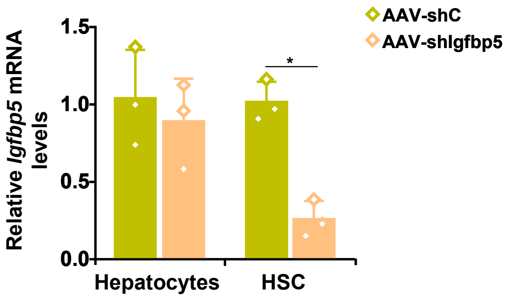

Fig.S16: C57/B6 mice were injected with AAV6 carrying shRNA under the control of the *Postn* promoter followed by injection with CCl_4 for 4 wk. Primary hepatocytes and HSCs were isolated and IGFBP5 expression was examined by qPCR.

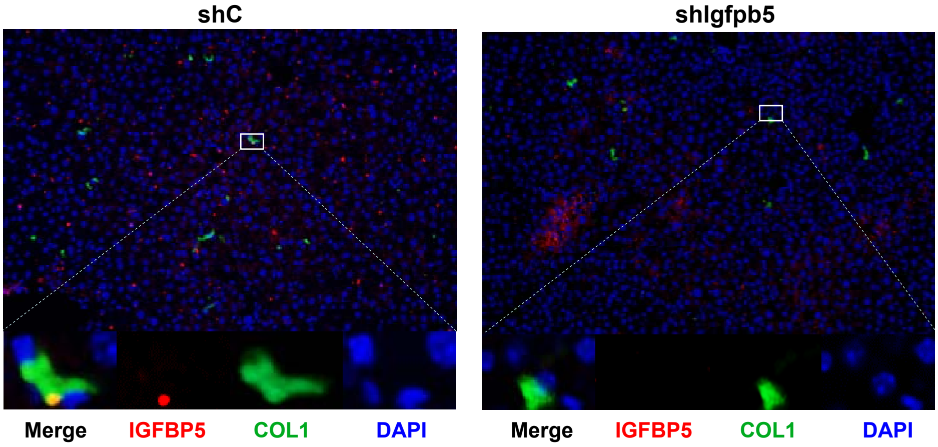

Fig.S17: C57/B6 mice were injected with AAV6 carrying shRNA under the control of the *Postn* promoter followed by injection with CCl_4 for 4 wk. Immunofluorescence staining was performed with indicated antibodies.

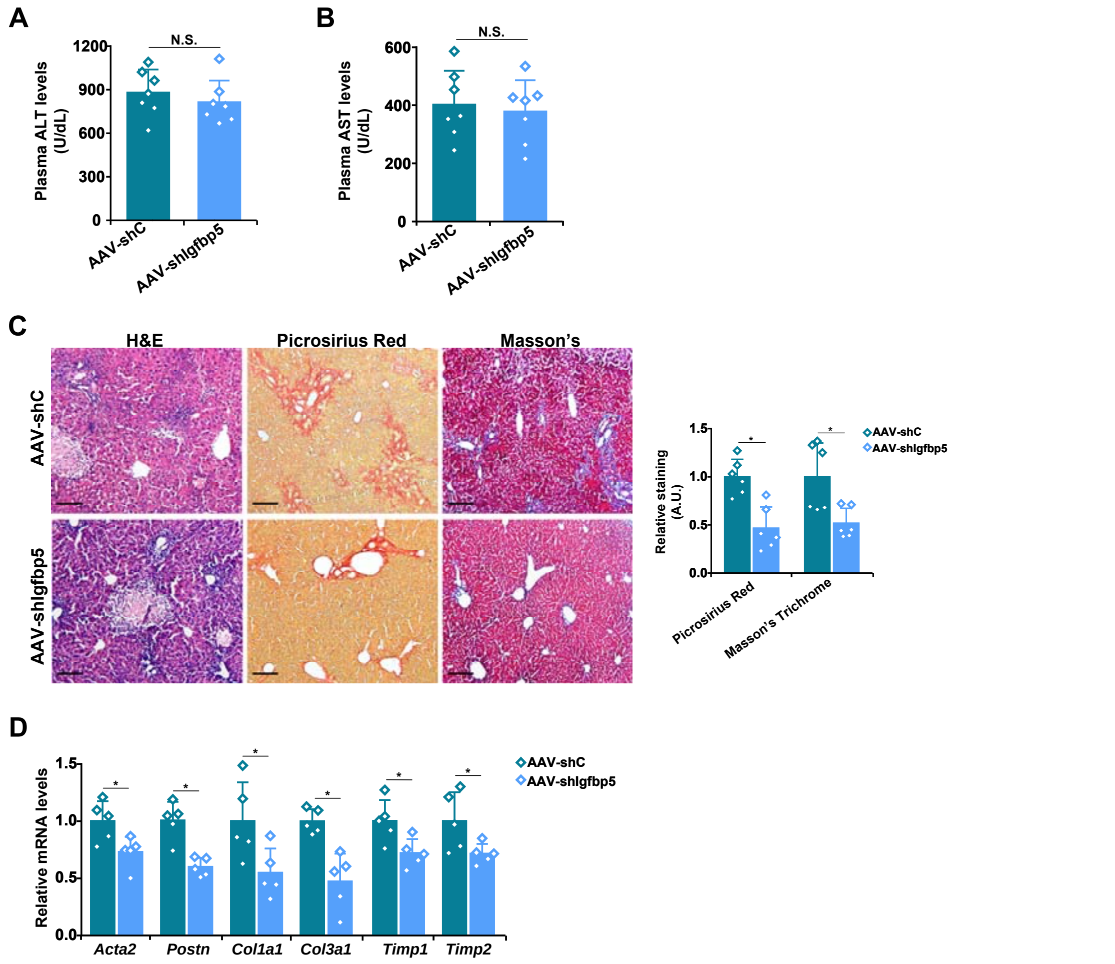

Fig.S18: (**A-D**) C57/B6 mice were injected with AAV6 carrying shRNA under the control of the *Postn* promoter followed by the BDL procedure. Plasma ALT levels (A). Plasma AST levels (B). H&E staining, picrosirius red staining, and Masson's trichrome staining (C). Myofibroblast marker gene expression levels were examined by qPCR (D).

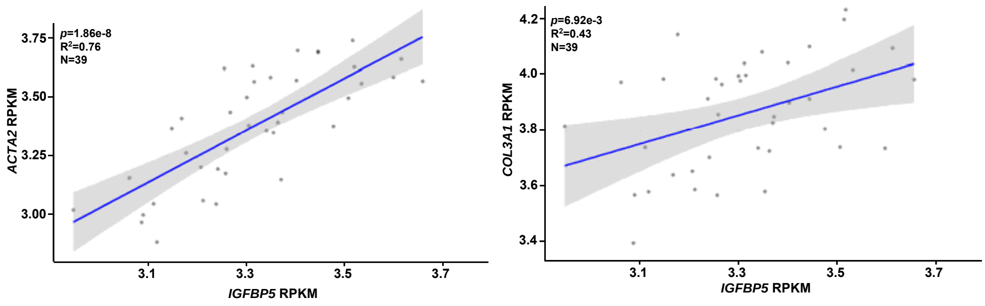

Fig.S19: Expression data for IGFBP5 and myofibroblast marker genes in human cirrhosis specimens were extracted from publicly deposited dataset (GSE139602). Linear correlation was performed with Graphpad.

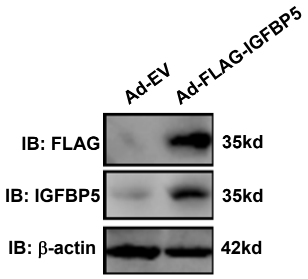

Fig.S20: Primary murine HSCs were transduced with adenovirus carrying a FLAG-tagged IGFBP5 vector or an empty vector (EV). IGFBP5 expression was verified by Western blotting.
